# Hepatocyte SGK1 activated by hepatic ischemia-reperfusion promotes the recurrence of liver metastasis via IL-6/STAT3

**DOI:** 10.1186/s12967-023-03977-z

**Published:** 2023-02-14

**Authors:** Xiangdong Li, Ziyi Wang, Chenyu Jiao, Yu Zhang, Nan Xia, Wenjie Yu, Xuejiao Chen, Likalamu Pascalia Wikana, Yue Liu, Linfeng Sun, Minhao Chen, Yuhao Xiao, Yuhua Shi, Sheng Han, Liyong Pu

**Affiliations:** 1 Hepatobiliary Center, The First Affiliated Hospital of Nanjing Medical University, Nanjing, China; 2 Key Laboratory of Liver Transplantation, Chinese Academy of Medical Sciences, Nanjing, China; 3 NHC Key Laboratory of Living Donor Liver Transplantation (Nanjing Medical University), Nanjing, China; 4 Department of General Surgery, Affiliated Yancheng School of Clinical Medicine of Nanjing Medical University, Yancheng, China

**Keywords:** Hepatic ischemia reperfusion, Tumor recurrence, SGK1, STAT3, NRF2

## Abstract

**Background:**

Liver metastasis is the leading cause of death in patients with colorectal cancer (CRC). Surgical resection of the liver metastases increases the incidence of long-term survival in patients with colorectal liver metastasis (CRLM). However, many patients experience CRLM recurrence after the initial liver resection. As an unavoidable pathophysiological process in liver surgery, liver ischemia-reperfusion (IR) injury increases the risk of tumor recurrence and metastasis.

**Methods:**

Colorectal liver metastasis (CRLM) mouse models and mouse liver partial warm ischemia models were constructed. The levels of lipid peroxidation were detected in cells or tissues. Western Blot, qPCR, elisa, immunofluorescence, immunohistochemistry, scanning electron microscope, flow cytometry analysis were conducted to evaluate the changes of multiple signaling pathways during CRLM recurrence under liver ischemia-reperfusion (IR) background, including SGK1/IL-6/STAT3, neutrophil extracellular traps (NETs) formation, polymorphonuclear myeloid-derived suppressor cell (PMN-MDSC) infiltration.

**Results:**

Hepatocyte serum/glucocorticoid regulated kinase 1 (SGK1) was activated in response to hepatic ischemia-reperfusion injury to pass hepatocyte STAT3 phosphorylation and serum amyloid A (SAA) hyperactivation signals in CRLM-IR mice, such regulation is dependent on SGK-activated IL-6 autocrine. Administration of the SGK1 inhibitor GSK-650394 further reduced ERK-related neutrophil extracellular traps (NETs) formation and polymorphonucler myeloid-derived suppressor cells (PMN-MDSC) infiltration compared with targeting hepatocyte SGK1 alone, thereby alleviating CRLM in the context of IR.

**Conclusions:**

Our study demonstrates that hepatocyte and immune cell SGK1 synergistically promote postoperative CRLM recurrence in response to hepatic IR stress, and identifies SGK1 as a translational target that may improve postoperative CRLM recurrence.

**Supplementary Information:**

The online version contains supplementary material available at 10.1186/s12967-023-03977-z.

## Background

Colorectal cancer (CRC) is one of the three most common cancers and ranks second in mortality due to cancer-related deaths [1]. Over the past three decades, the number of cases and deaths from colorectal cancer worldwide has more than doubled [2]. In China, the incidence and mortality rates of CRC are ranked third [3]. It is estimated that by 2022, there will be 592,232 new cases of colorectal cancer and 309,114 new deaths in China [4]. The incidence in developing countries is one-fourth of that in developed countries, and this trend is likely to continue as the Human Development Index increases [1].

Surgical resection of the primary tumor offers patients with CRC the best chance of long-term disease-free survival. Unfortunately, a large proportion of CRC patients develop metastases even after successful resection of the primary tumor [5]. During liver surgery, the hepatic hilum is often clamped to severe blood loss and the need for blood transfusion, [6, 7] resulting in ischemic injury to hepatocytes, and hyperactivation of inflammatory pathways following restoration of blood flow can lead to further dysfunction and damage. This process, known as ischemia reperfusion (IR) injury, is an unavoidable consequence of liver resection and is a major cause of morbidity and mortality [8, 9]. IR indury during liver resection has been shown to accelerate liver cancer progression, while severe residual liver ischemia is associated with reduced cancer-specific survival rates [10]. IR injury during liver surgery has been shown to accelerate the progression of colorectal liver metastases (CRLM) in animal models [11–14].

It is generally accepted that the signaling events that lead to local hepatocyte injury by IR are complex and diverse, involving hepatocytes, hepatic sinusoidal endothelial cells (LSEC), Kupffer cells (KC), hepatic stellate cells (HSC), and infiltrative cells and the synergy of neutrophils, macrophages, platelets [15].

Ischemic injury is a local process of cellular metabolic disturbance caused by glycogen depletion, insufficient oxygen supply, and ATP depletion, which lead to initial parenchymal cell death. DAMPs released from cell death, tissue injury-induced complement activation, and oxygenation-triggered mitochondrial reactive oxygen species (ROS) production all contribute to hepatic immune activation after reperfusion [16]. Therefore, hepatocytes are very negatively affected by liver IR injury during liver surgery. An increasing number of studies have shown that hepatocytes are the main driver of CRLM. Human and murine colorectal cancer cells induce apoptosis in hepatocytes, thereby creating a niche for tumor growth [17]. Studies have shown that the activation of STAT3 in hepatocytes and the overexpression of SAA form a pro-metastatic niche that promotes liver metastasis of pancreatic and colorectal cancers [18]. In cases of pancreatic cancer liver metastases, significantly elevated levels of circulating SAA in patients were associated with reduced survival rates [19].

Furthermore, neutrophil activation is closely associated with IR-related liver parenchymal injury [15]. In addition to their well-known function in the acute phase of the immune response, neutrophils also play an important role in various stages of tumor initiation and progression by releasing their stored or newly synthesized mediators. In addition to reactive oxygen species, cytokines, chemokines, granulin, and lipid mediators, neutrophil extracellular traps (NETs) can also be released after neutrophil activation [20]. NETs are networks of granulin and decondensed chromatin, which capture and kill pathogens during infection [21, 22]. NETs released by neutrophils during infection to capture microorganisms have been implicated in cancer metastasis in mouse models [23]. In a mouse CRLM model, neutrophils formed NETs in response to surgical stress to promote the progression of liver metastases [13, 24]. In multiple mouse models, NETs have been reported to recruit tumor cells to pre-metastatic niches via CCDC25 [25]. In addition, unlike neutrophils or monocytes activated through the DAMP and PAMP pathways, the long-term presence of myeloid growth factors and inflammatory signals in cancer, chronic infection or inflammation, autoimmune disease settings, and immune activation of the pathological state caused by the continuous stimulation of the myeloid cell compartment will form the myeloid-derived suppressor cell MDSC, which has a strong immunosuppressive activity. MDSCs mainly include two types: M-MDSCs and PMN-MDSCs [26]. MDSCs promote pre-metastatic niche formation by inducing angiogenesis and tumor cell invasion, and suppressing antitumor immune responses [27, 28].

Serum and glucocorticoid-induced protein kinase 1 (SGK1) is a member of the “AGC” subfamily of protein kinases, which shares structural and functional similarity with the AKT kinase family, and shows serine/threonine kinase activity. SGK1 activation is involved in pathophysiological processes including inflammation and tumorigenesis [29, 30]. In a mouse model, administration of an SGK1 inhibitor (GSK-650394) reduced cerebral ischemia–reperfusion injury [31, 32]. In a rat model of global cerebral ischemia, administration of GSK-650394 was noted to improve the learning and memory deficits caused by cerebral ischemia in rats [33]. SGK1 plays an important role in tumorigenesis, proliferation, apoptosis, invasion, metastasis and autophagy processes [30]. In mouse models, activation of neutrophil SGK1 promotes ROS production and NETs formation [34].

We explored whether SGK1 activated by hepatic IR could affect the postoperative recurrence of CRLM in murine models and the corresponding mechanism by specifically targeting hepatocytes or by administering pharmacological inhibitors. Our results suggest that hepatocyte SGK1 is dependent on the IL-6/stat3/SAA axis to promote CTC colonization after IR. Further extending our study revealed that SGK1 anchored to PMN-MDSCs is necessary for NETs formation and PMN-MDSC differentiation and activity after IR in the context of CRLM. Overall, SGK1 may be a promising target for ameliorating postoperative CRLM recurrences.

## Methods

### Clinical patients and animals

Biopsy specimens were obtained from 5 patients with benign liver disease and 16 patients with CRLM who underwent partial hepatectomy (PHY) with the Pringle maneuver [35]. Peripheral blood samples were analyzed for preoperative and postoperative serum alanine aminotransferase (sALT) and aspartate aminotransferase (sAST) levels. Hepatic biopsies were obtained after reperfusion (before abdominal closure). The ischemia time was between 15 and 30 min. All the participants provided informed consent. All patients who donated samples for this project signed an informed consent form, and all experiments were approved by the ethics committee of Nanjing Medical University.

### Mouse Liver partial warm ischemia models, metastases models

6–8 weeks old C57/BL6 male mice purchased from Vital River Laboratory Animal Technology Co. Ltd (Beijing, China) were housed under standard laboratory conditions. They were provided with adequate food and water, ambient relative humidity of 50–60%, controlled temperature of 22–26 °C and a light/dark cycle of 12 h. All the animal experiments were approved by the Institutional Animal Care and Use Committee (IACUC) of Nanjing Medical University (IACUC-2211025).

The mice were subjected to partial liver warm ischemia, followed by 3 h to 2 days of reperfusion [36]. Briefly, under isoflurane anesthesia, a midline laparotomy was performed to expose the liver. The mice were then injected with heparin (100U/kg), and an atraumatic clip was used to interrupt both the arterial and portal venous blood supply to the cephalad-liver lobes. After 90 min of partial hepatic ischemia, the clip was removed to initiate the hepatic reperfusion. Sham-operated mice underwent the same procedure but without vascular occlusion. Mice were euthanized after 3 h to 2 days after of reperfusion to obtain liver and serum samples.

Colorectal liver metastases were induced in mice as previously described [37]. In brief, 1 × 10^6^ MC38 or MC38/Luc cells (Qiaoyuan Biotech, Nanjing, China) in 100 μl PBS were injected through a 3 cm midline laparotomy into the spleen of 8–12 weeks old C57BL/6 J WT mice using a 28G insulin syringe. Tumor cells were allowed to circulate for 15 min. Mice that underwent IR were subjected to a nonlethal model of segmental (70%) hepatic warm ischemia (90 min) and reperfusion 15 min after splenectomy. Splenectomy was performed to prevent the formation. An increase in the number of liver metastases was observed in the ischemic lobes within 2 weeks of reperfusion.

### Hepatocellular function assay

sALT and sAST levels were measured using corresponding kits (S03030, S03040) to export the results after the automatic biochemical analyzer (Rayto Life and Analytical Sciences Co. Ltd. Chemray 240).

### Histology, immunohistochemistry, and immunofluorescence staining

The specimens were fixed in 4% neutral buffered formalin and then embedded in paraffin. 4 μm-thick Liver sections were stained with hematoxylin and eosin. The severity of liver IR was graded using Suzuki’s criteria on a scale from 0 to 4, or with antibodies using standard immunohistochemistry protocols. For Immunofluorescence, the fixed tissue sections were placed in a repair kit (Servicebio, China) filled with EDTA antigen retrieval buffer (PH = 9.0), and then boiled in a microwave for repair. After natural cooling, the slides were washed three times with PBS. BSA (3%) in PBS solution was added for 30 min to block nonspecific binding, and then the corresponding primary antibodies were added: anti-SGK1(23394–1-AP, Proteintech), anti-p-STAT3 (Tyr705) (bs-1658R, Bioss), anti-HNF-4α (ab201460, Abcam), anti-IL-6 (Servicebio, China), anti-MPO (ab208670, Abcam), anti-CitH3 (ab281584, Abcam), anti-CD36 (Servicebio, China), anti-Ly6G (Servicebio, China), and anti-SAA (Servicebio, China). After overnight incubation, the secondary antibody against the corresponding species was added for 50 min in the dark. DAPI was used for the nuclear counterstaining. Slides were observed under a confocal fluorescence microscope (NIKON ECLIPSE C1) [35].

### TUNEL assay

TUNEL staining was conducted using a Tunel kit, according to the manufacturer’s protocols using freshly frozen tissues. (Servicebio, China) The apoptotic cells were determined and quantified by counting the positive cells in 4 fields with at least 300 cells per field in each group.

### Hepatocytes and nonparenchymal cells (NPC) isolation

Hepatocytes were isolated from normal or IR-livers of B6 mice by in situ collagenase perfusion [38]. Briefly, livers were perfused via the portal vein with HBSS supplemented with 2% heat-inactivated FBS, followed by 0.27% collagenase IV (Sigma, St Louis, MO, USA). Perfused livers were dissected and teased through 70 μm nylon mesh cell strainers (BD Biosciences, San Diego, CA, USA). Hepatocytes were separated by centrifuging at 50 g for 2 min three times. NPC were separated by collecting the supernatant and then centrifuging at 300 g for 10 min.

### In vivo* siRNA knockdown*

Mix SGK1 siRNA or control siRNA (Sangon Biotech, Shanghai, China) and galactose-conjugated [39] polymer (polyplus-transfection SA, Illkirch, France) according to the manufacturer's instructions were injected 4 h before performing IR surgery on mice through the tail vein (siRNA 200 mg/kg) to achieve relative specific anchorage of hepatocytes. In vivo-jetPEI®-Gal corresponds to galactose-conjugated in vivo-jetPEI® designed to enhance in vivo transfection of cells expressing galactose-specific membrane lectins, such as hepatocytes bearing the asialoglycoprotein receptor (ASGP-R or Gal/GalNAc receptor).

### Cell culture and hypoxia-reoxygenation (H/R) model

Immortalized normal human hepatocyte-derived liver cells (LO2), or mouse liver cell line AML-12 from Hepatobiliary Center of The First Affiliated Hospital of Nanjing Medical University were cultured in DMEM-F12 containing 10% fetal bovine serum in an incubator with 5% CO2 at 37 °C. The cells were passaged on the second day, and those in the logarithmic growth phase were harvested for the experiments.

For the H/R model, cells were first cultured in serum-free medium for 24 h, and then they were transferred into a hypoxic incubator with 1% O_2_, 5% CO_2_, and 94% N_2_ for another 6 h. After the hypoxia treatment, the cells were transferred into a normoxic incubator (21% O_2_, 5% CO_2_, and 74% N_2_) with fresh culture medium supplemented with 10% FBS, and cultured for 1 h as previously described [40].

For the reactive oxygen species (ROS) assay, the ROS in LO2 or AML cells were measured using dichloro-dihydro-fluorescein diacetate (DCFH-DA) according to the manufacturer’s instructions. Briefly, cells were incubated with 10 μM DCFH-DA (S0033, Beyotime, Shanghai, China) for 10 min, then the cells were washed with serum-free medium three times, and subjected to flow cytometry analysis (CytoFLEX S, Beckman). All the data were analyzed using FlowJo (V10.8.1) and CytoExpert (V2.4.0.28).

For the apoptosis detection, an apoptosis detection kit (640932, biolegend) was used according to the manufacturer’s instructions. Cells undergoing H/R were washed twice with cold BioLegend’s Cell Staining Buffer (420201,biolegend), and then resuspend cells in Annexin V Binding Buffer at a concentration of 0.25–1.0 × 107 cells/mL. 100 µL of cell suspension was transferred in a 5 mL test tube, and then 5 µL of APC Annexin V and 10 µL of Propidium Iodide Solution was added to the tube for 15 min at room temperature (25 °C) in the dark. Finally, 400 µL of Annexin V Binding Buffer was added to each tube before analysing by flow cytometry with proper machine settings.

### Bioluminescent imaging

Bioluminescent imaging was used to monitor tumor burden at 7 days in a nonlethal manner. Prior to imaging, mice were anesthetized using Isofluorane followed by i.p. injection of D-Luciferin, Potassium Salt (Maokang Biotech, Shanghai, China) at a concentration of 150 mg/kg in 0.1 ml PBS 10 min. The mice were then placed in the chamber for an IVIS Spectrum imaging system (PerkinElmer, USA) from Nanjing Medical University.

### Human/Mouse neutrophils isolation and culture

Mouse neutrophils were isolated using the EasySep Mouse Neutrophil Enrichment Kit, according to the manufacturer’s instructions (STEMCELL Technologies) [41]. Human neutrophils were isolated using the EasySep Direct Human Neutrophil Isolation Kit (STEMCELL Technologies), according to the manufacturer’s protocol [42]. Isolated neutrophils were resuspended in RPMI 1640 containing 2% fetal bovine serum (FBS).

### In vitro* NETs formation*

Neutrophils were stimulation with 100 nM phorbol 12-myristate 13-acetate (PMA) or 100 ng/ml Lipopolysaccharide (LPS) for 3 h at 37 °C. Cells were fixed with 2% paraformaldehyde and incubated overnight at 4 °C. Immunofluorescence was performed to detect the NETs marker citrullinated histone3 (CitH3). Briefly, cells were washed three times with PBS, permeabilized with 0.1% Triton X-100 and 0.1% sodium citrate for 10 min at 37 °C, and then blocked with 3% bovine serum albumin for 90 min at 37 °C. Cells were incubated with 1:1000 rabbit anti-CitH3 (ab281584, Abcam) overnight at 4 °C and then with 1:1000 Alexa 488 donkey anti-rabbit immunoglobulin G (ab150073, Abcam) for 2 h at room temperature.

### Scanning electron microscope observations

As described above, stimulated neutrophils were fixed for 24 h at 4 °C with 2.5% glutaraldehyde. After standard dehydration and sputter coating process, the NETs were visualized under a scanning electron microscope (JSM-7900F, JEOL) at an acceleration voltage of 2.0 kV from Nanjing Medical University.

### Flow cytometry

Isolated hepatocytes or neutrophils were fixed with 4% paraformaldehyde solution at 4 °C for 10–20 min according to the manufacturer’s instructions (554714, BD Biosciences), and the cells were resuspended in BD Perm/Wash™ buffer for 15 min. After the cells were resuspended in 50 µL of BD Perm/Wash™ buffer containing a pre-determined optimal concentration of a fluorochrome-conjugated anti-cytokine antibody: anti-PE-CD84 (122805, biolegend), anti-BV421-Ly6G (12762, biolegend), anti-Fitc-LOX1 (ab81710, Abcam) or appropriate negative control (400625, biolegend) and incubated at 4 °C for 30 min in the dark, the cells were washed 2 times with 1 × BD Perm/Wash™ buffer (1 mL/wash for staining in tubes and 250 µL/wash final volume for staining in microwell plates) and were resuspended in staining buffer for flow cytometric analysis (CytoFLEX S, Beckman). All data were analyzed using FlowJo (V10.8.1) and CytoExpert (V2.4.0.28).

### Quantitative RT-PCR

After extraction from tissues or cells, total RNA (2.0 μg) was reverse-transcribed into cDNA using the SuperScriptTM III First-Strand Synthesis System (Invitrogen, Carlsbad, CA). Quantitative-PCR was performed using a DNA Engine with Chromo 4 Detector (MJ Research, Waltham, MA, USA). In a final reaction volume of 20 μl, the following were added: 1xSuperMix (Platinum SYBR Green qPCR Kit, Invitrogen, Carlsbad, CA), cDNA and 0.5 mM of both forward and reverse primers. Amplification conditions were: 50 °C (2 min), 95 °C (5 min) followed by 50 cycles of 95 °C (15 s), 60 °C (30 s). Primer sequences used for amplification are listed in Additional file 1: Table. All expression levels and results of the target genes were standardized for the β-actin expression.

### ELISA assay

Murine serum and cell-free culture supernatants were collected. And then the ELISA kit (Thermo Fisher Scientific) was used to measure cytokine levels.

### Western blot

Tissues or cells were lysed with RIPA buffer, separated by SDS-PAGE gel electrophoresis and then transferred to a PVDF membrane. The proteins were then immunoblotted with the primary antibody: anti-SGK1 (bms-33275 M, Bioss), anti-pSTAT3 (bs-16558R, Bioss), anti-STAT3 (ab109085, Abcam), anti-NRF2 (bs-1074R, Bioss), anti-ERK1 + ERK2 (ab184699, Abcam), anti-pERK1 + pERK2 (ab214036, Abcam), anti-β-actin (4970, Cell Signaling Technology), anti-MPO (ab208670, Abcam), anti-CitH3 (ab281584, Abcam), anti-NE (bs-6982R, Bioss), anti-PAD4 (ab214810, Bioss). This was followed by color development using horseradish peroxidase (HRP)-enhanced chemiluminescence (ECL) assays.

### Statistical analysis

Representative results are shown, and data are presented as the mean ± SD. Statistical significance was analyzed using the Student’s unpaired t test. Linear regression (R2) was used to evaluate the strength of linear relationship between variables. Two-tailed P values less than 0.05 were considered statistically significant.

## Results

### Hepatocyte SGK1 expression is up-regulated in response to hepatic ischemia reperfusion

**Fig. 1:**
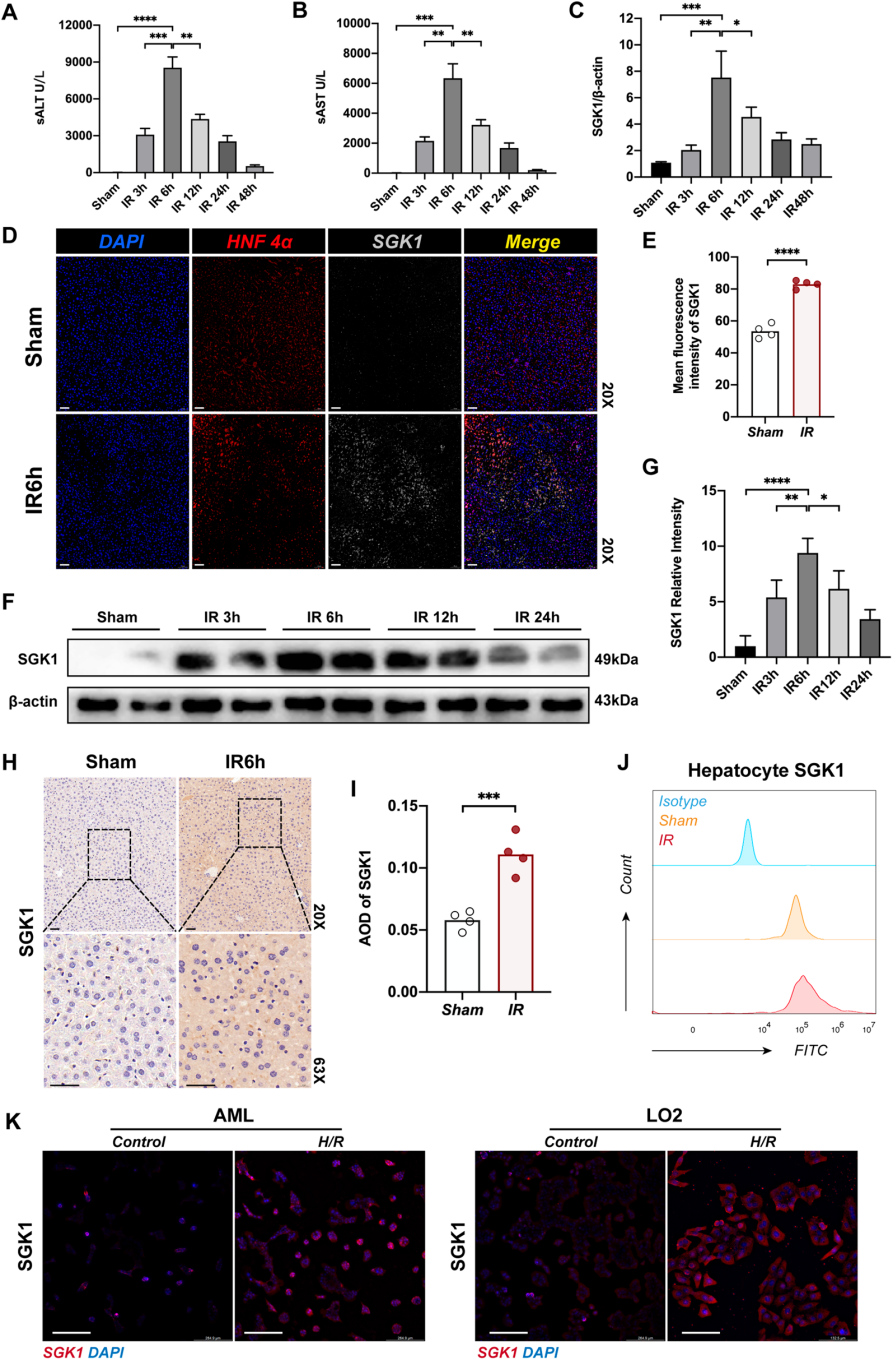
Hepatocyte SGK1 expression is up-regulated in response to hepatic ischemia reperfusion. 6–8 weeks mice were first subjected to 70% liver warm ischemia, followed by reperfusion for 3, 6, 12, 24, and 48 h. **A** and **B** Liver function was evaluated by sALT and sAST levels (U/L) (n = 3 samples/group). **C** Quantitative RT-PCR of SGK1 in ischemic livers or control group livers (n = 4 samples/group). **D** Immunofluorescence staining of SGK1 in ischemic livers or control livers, scale bars, 50 μm (n = 4 samples/group). **E** Mean fluorescence intensity of SGK1 (n = 4samples/group). **F** The expression of hepatocytes SGK1 was detected by western blotting (n = 4 samples/group). **G** Western blot analysis and relative intensity of SGK1 in hepatocytes (n = 4 samples/group). **H** Immunohistochemistry staining of SGK1 in ischemic livers or sham livers, scale bars, 50 μm. (n = 4 samples/group). **I** Quantification of hepatic SGK1 immunohistochemistry (n = 4 samples/group). **J** SGK1 expression in primary hepatocytes isolated from ischemic or control livers were detected by flow cytometry (n = 3 samples/group). **K** Detection of SGK1 expression in LO2 and AML cell lines after H/R by immunofluorescence (n = 3 samples/group). All data represent the mean ± SD. *p < 0.05, **p < 0.01, ***p < 0.001, ****p < 0.0001

To simulate ischemia-reperfusion injury (IRI) of the liver caused by hepatic portal blockade during liver surgery, we performed warm ischemia in mice for 90 min, followed by reperfusion for 3, 6, 12, 24, and 48 h. We found that sALT and sAST levels were relatively highest in the liver at IR6h (Fig. 1A, B). Compared to the control group, the transcript level of SGK1 in the ischemic liver lobe was higher (Fig. 1C), and the protein expression of SGK1 in the ischemic liver lobe was also higher (Fig. 1D, E, H and I). The expression of SGK1 in the IR liver was mainly localized on HNF-4α-positive cells (Fig. 1D) [43], suggesting that in IR, it is mainly hepatocyte SGK1, which is activated in response to the induction of inflammation by hepatic IR. We isolated primary hepatocytes from IR livers and found that SGK1 was up-regulated on hepatocytes (Fig. 1F, G and J). In vitro, we performed H/R assays with the human hepatocyte cell line LO2 or mouse hepatocyte cell line AML12, and found that SGK1 signal was expectedly activated by H/R (Fig. 1K).

### Absence of hepatocyte SGK1 results in hepatic IR alleviation

**Fig. 2:**
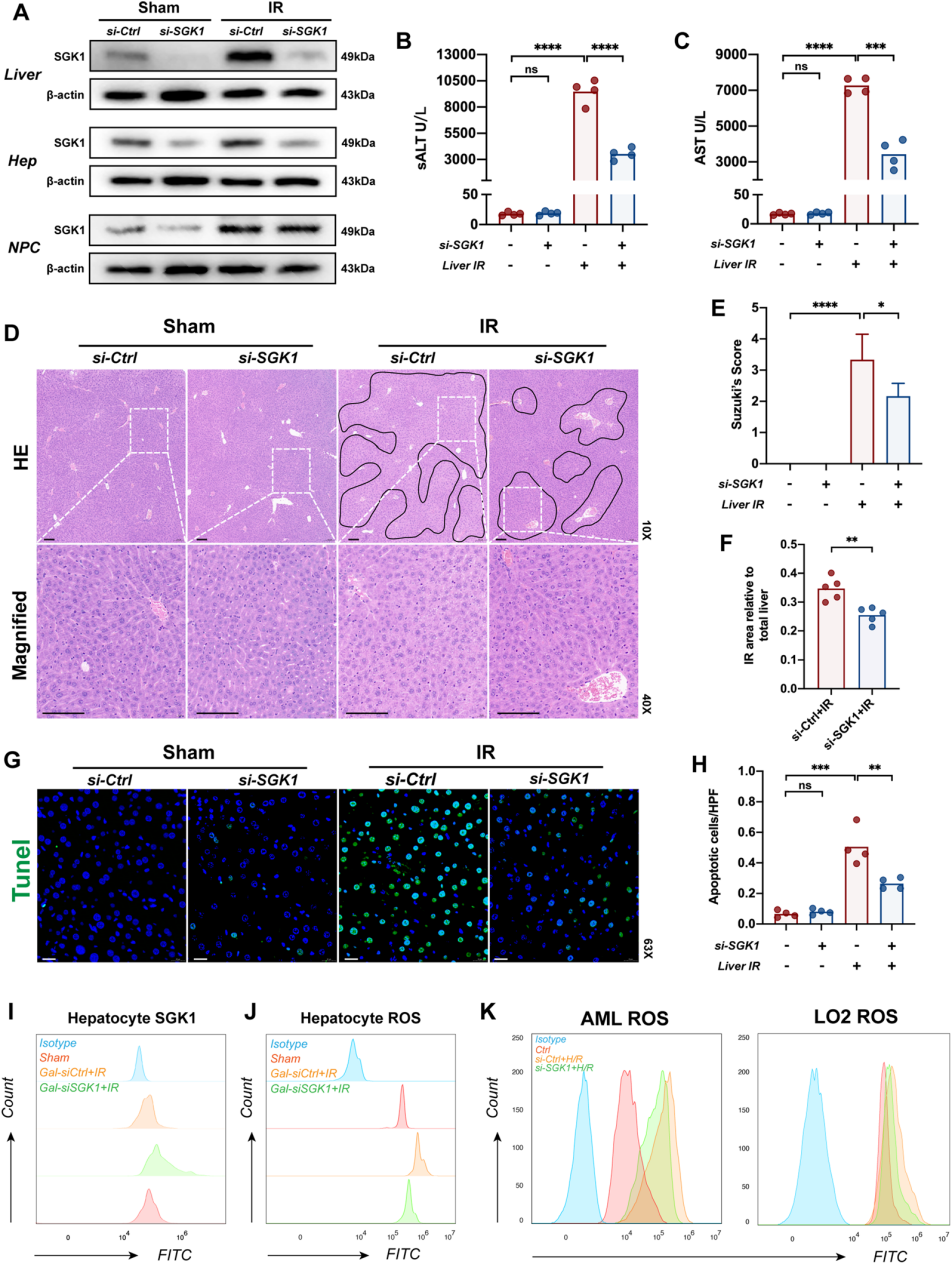
Absence of hepatocyte SGK1 results in hepatic IR alleviation. 6–8 weeks wild type mice were first subjected to 70% warm ischemia, followed by reperfusion for 6 h. **A** Using Gal-siSGK1 or Gal-siCtrl, and the SGK1 expression was detected by western blot in primary hepatocytes or nonparenchymal cells isolated from ischemia livers or sham livers. SGK1 expression was also detected in ischemic liver tissue or sham liver tissue by western blot (n = 3 samples/group). **B** and **C** Liver function was evaluated by sALT and sAST levels (IU/L) (n = 3 samples/group). **D** Representative histological staining (H&E) of ischemic liver tissue (n = 6 samples/group). Scale bars, 100 μm. **E** Suzuki's histological score (n = 6 samples/group). **F** IR area calculated by Caseviewer (n = 5samples/group). **G** Apoptosis of liver cells was detected by tunel staining (n = 4 samples/group). Scale bars, 20 μm. **H** Quantification of apoptosis cells (n = 4 samples/group). **I** SGK1 expression of primary hepatocytes isolated from ischemic or sham livers was detected by flow cytometry (n = 3 samples/group). **J** ROS expression of primary hepatocytes isolated from ischemic or sham livers was detected by flow cytometry (n = 3samples/group). **K** Detection of ROS activation in LO2 and AML cell lines after H/R by flow cytometry (n = 3samples/group). All data represent the mean ± SD. *p < 0.05, **p < 0.01, ***p < 0.001, ****p < 0.0001

To better clarify the role of hepatocyte SGK1 in regulating inflammation during hepatic IR, we used a galactose-conjugated linear polyethyleneimine derivative (polyplus-transfection SA, Illkirch, France) as a carrier to coupled SGK1-siRNA, which was transported the complex to hepatocytes through the tail vein 4 h before IR surgery (40 μg/mouse, IV, repeated twice). The efficiency of knockdown targeting hepatocytes rather than nonparenchymal cells (NPC) in vivo was verified using western blotting or flow cytometry (Fig. 2A and I). We found that SGK1 inhibition in hepatocytes alleviated liver damage (Fig. 2B, C). Gal-siSGK1-pretreated mice exhibited less hepatocyte edema, sinusoidal congestion, and necrosis than those in the control group (Fig. 2D–F). Furthermore, apoptosis of hepatocytes in response to IR was reduced by Gal-siSGK1 treatment in vivo (Fig. 2G, H). As predicted, treatment with Gal-siSGK1 reduced IL-1β, TNF-α, iNOS, CXCL2 and CXCL10 mRNA expression in the ischemic liver (Additional file 2: Fig. S1A), accompanied by decreased levels of IL-1β in the serum (Additional file 2: Fig. S1B). Surprisingly, we found that SGK1 deficiency reduced ROS activation in mouse primary hepatocytes after IR, which has been shown to play an important role in driving hepatic IR [44] (Fig. 2J). In vitro, we performed H/R assays with LO2 or AML12 cell lines and found that ROS production was expectedly inhibited by Gal-siSGK1 (Fig. 2K). Administration of Gal-siSGK1 reduces apoptosis in AML and LO2 cells undergoing H/R (Additional file 2: Fig. S2A, B).

### Targeting hepatocyte SGK1 alleviates IR-driven colorectal cancer liver metastasis

**Fig. 3:**
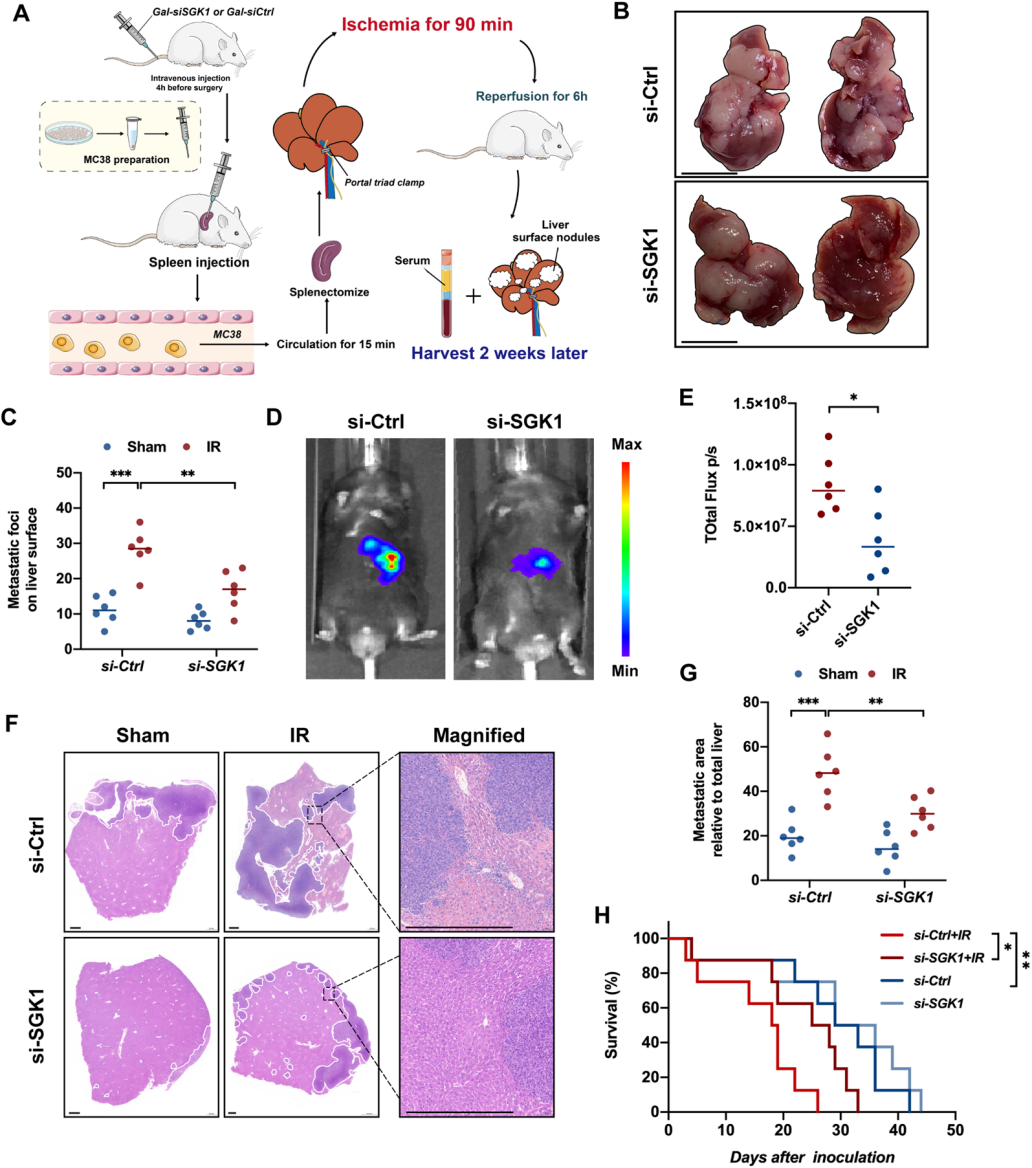
Targeting hepatocyte SGK1 alleviates IR-driven colorectal cancer liver metastasis. **A** A schematic representation of the experimental design is depicted. We injected SGK1-siRNA mannose-conjugated complex into the tail vein of mice to knock down hepatocytes SGK1 at first. Intrasplenic injection of luciferase-labeled MC38 colorectal cancer cell line was then performed. After 15 min circulation, mice were subjected to liver IR and a splenectomy at the same time. **B** Representative image of CRLM (n = 6 samples/group). **C** The number of metastases on liver surface (n = 6 samples/group). **D** and **E** Representative image of bioluminescence and statistics were shown (n = 6 samples/group). **F** Representative histological staining (H&E) of ischemic liver tissue (n = 4samples/group). Scale bars, 500 μm. **G** Tumor area (n = 6 samples/group). **H** Survival (n = 8samples/group). All data represent the mean ± SD. *p < 0.05, **p < 0.01, ***p < 0.001, ****p < 0.0001

To determine whether SGK1 knockdown in hepatocytes affects the burden of CRLM, we used a highly standardized murine model of liver metastasis. The mice were subjected to a splenectomy together with partial (70%) hepatic I/R 15 min after the intrasplenic injection of luciferase-labeled MC38 murine CRC cells (MC38) (Fig. 3A). We found that inhibition of SGK1 in hepatocytes significantly reduced the progression of liver metastatic nodules after splenic injection and IR (Fig. 3B, C). As expected, the size of the metastasis was significantly reduced (Fig. 3F, G). Furthermore, knockdown of hepatocyte SGK1 significantly slowed IR-related tumor growth (Fig. 3D, E) and prolonged the survival of mice (Fig. 3H).

### The Progression of CRLM-IR regulated by hepatocytes SGK1 depends on the activation of STAT3

**Fig. 4:**
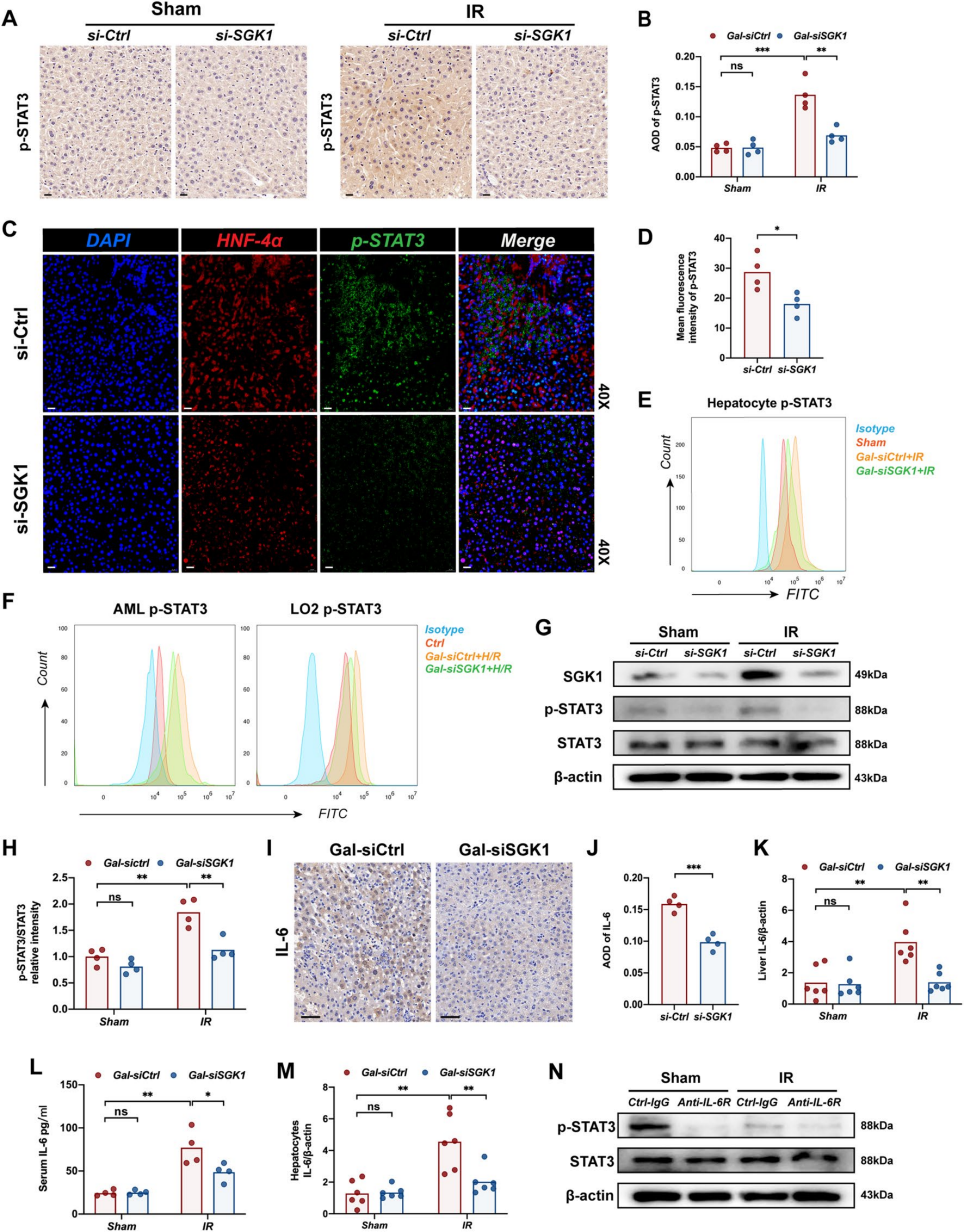
The Progression of CRLM-IR regulated by hepatocytes SGK1 depends on the activation of STAT3. Using Gal-siSGK1or Gal-siCtrl and then a 90-min warm ischemia model was constructed in mice, and the liver was collected after 6 h reperfusion. **A** Immunohistochemical staining of p-STAT3 expression in ischemic livers or sham livers (n = 4 samples/group). Scale bars, 20 μm. **B** Quantification of p-STAT3 immunohistochemistry (n = 4 samples/group). **C** Immunofluorescence staining of p-STAT3 and HNF-4α expression in ischemic livers or sham livers (n = 4 samples/group), scale bars, 20 μm. **D** Quantification of p-STAT3 immunofluorescence (n = 4 samples/group). **E** Expression of p-STAT3 in primary hepatocytes was detected by flow cytometry (n = 3samples/group). **F** Flow cytometry detection of p-STAT3 expression in AML and LO2 after H/R (n = 3 samples/group). **G** The expression of SGK1, p-STAT3 and STAT3 in ischemic livers or sham livers was detected by western blot (n = 4 samples/group). **H** Quantification of p-STAT3/STAT3 relative intensity (n = 4 samples/group). **I** Immunohistochemistry staining for IL-6 expression in ischemic livers or control livers (n = 4 samples/group), scale bars, 50 μm. **J** Quantification of IL-6 immunohistochemistry (n = 4 samples/group). **K** Detection of mRNA levels of IL-6 in ischemic liver tissue or sham liver tissue by Quantitative RT-PCR (n = 6 samples/group). **L** The level of serum IL-6 in mice was detected by Elisa (n = 4 samples/group). **M** Detection of mRNA levels of IL-6 in hepatocytes isolated from ischemic liver lobes by Quantitative RT-PCR (n = 6 samples/group). **N** Western Blot detection of p-STAT3 and STAT3 in ischemic livers or sham liver treated with IL-6R antibody or control antibody (n = 3 samples/group). All data represent the mean ± SD. *p < 0.05, **p < 0.01, ***p < 0.001, ****p < 0.0001

STAT3 signaling has been proved great contribution to liver injury in mice [45]. Relative research shows SGK1 is a positive regulator of STAT3 in HEK293 cells [46]. We subsequently detected the activation of STAT3 signaling in mouse ischemic liver lobes. However, STAT3 activation was reversed in both livers and isolated hepatocytes of Gal-siSGK1 mice (Fig. 4A–E,G,H). In vitro, we also detected activation of STAT3 signaling in two hepatocyte lines, LO2 and AML, which underwent hypoxia-reoxygenation, and was reversed by Gal-siSGK1 (Fig. 4F). This demonstrates an intrinsic link between SGK1 and STAT3 phosphorylation in hepatocytes. Actually the application of the STAT3 inhibitor—Stattic [47] attenuated hepatic IR (Additional file 2: Fig. S3B–E) and the progression of CRLM after IR (Additional file 2: Fig. S3F–I). A previous study has demonstrated that hepatocytes express IL-6 in response to LPS in vitro and in vivo [48]. Hepatocytes regulate liver injury, repair, and inflammation in liver diseases by producing IL-6 [49]. IL-6 is the main activator of liver STAT3 [50]. We therefore wondered whether the regulation of STAT3 by SGK1 was dependent on IL-6. The administration of Gal-siSGK1 decreased the expression of IL-6 in livers of mice undergoing IR (Fig. 4I–K), and the level of serum IL-6 was also down-regulated (Fig. 4L). The expression of IL-6 was significantly increased in the hepatocytes isolated from ischemic livers (Fig. 4M). STAT3 phosphorylation was inhibited in ischemic livers treated with an antibody against IL-6R (BioXCell) after I/R, and p-STAT3 expression in the livers co-administered with Gal-siSGK1 was not further reduced (Fig. 4N). This shows that the phosphorylation of STAT3 depends on the regulation of IL-6 expression by SGK1.

The latest study found that SAA, a type of protein activated in the acute inflammatory period [51] was overexpressed in the hepatocytes and circulation of patients with CRLM, and confirmed in mouse models that SAA overexpressed in hepatocytes and circulation was dependent on IL-6/STAT3 signaling activation in non-malignant liver cells [18]. Combined with our previous studies, we hypothesized that the activation of SGK1/IL-6/STAT3 signaling in murine hepatocytes in response to hepatic IR, mediates the expression of SAA before CRC liver metastases. Compared to healthy livers from patients with benign liver disease, paratumor tissue from patients with CRLM expressed more SAA (Additional file 2: Fig. S4A, B). The level of SAA1/2 mRNA was significantly increased after IR surgery in the livers of mice with CRLM (Additional file 2: Fig. S4C). Interestingly, a specific knockout of SGK1 in hepatocytes by using Gal-siSGK1 reduced serum SAA levels in mice (Additional file 2: Fig. S4C).

It has been shown that Nuclear factor E2-related factor 2 (NRF2) contributes to chemoresistance [52] and SGK1 is an antioxidative factor that promotes NRF2’s activity. [53] We then investigated whether there was crosstalk between SGK1 and NRF2 in hepatocytes of mice with CRLM under the background of IR. In contrast of significantly higher expressed of SGK1 in HNF4α + hepatocytes in response to IR (Additional file 2: Fig. S5C), the expression level of NRF2 in ischemic hepatocytes from control group had no significant difference compared with that from Gal-siSGK1group (Additional file 2: Fig. S5A–C). Some studies showed that macrophages NRF2 was involved in the regulation of liver ischemia reperfusion injury [54]. Indeed, NRF2 seemed to mainly expresses on liver non-parenchymal cells rather than hepatocytes under I/R or CRLM-I/R background (Additional file 2: Fig. S5C–F). Futhermore, we found that the NRF2 mainly expressed in cancer cells of metastatic niche (Additional file 2: Fig. S5E, F). Therefore, we confirmed that liver metastasis regulated by hepatocyte SGK1 is independent of NRF2 crosstalk.

### GSK-650394 regulates NETs formation in CRLM mice with IR background

**Fig. 5:**
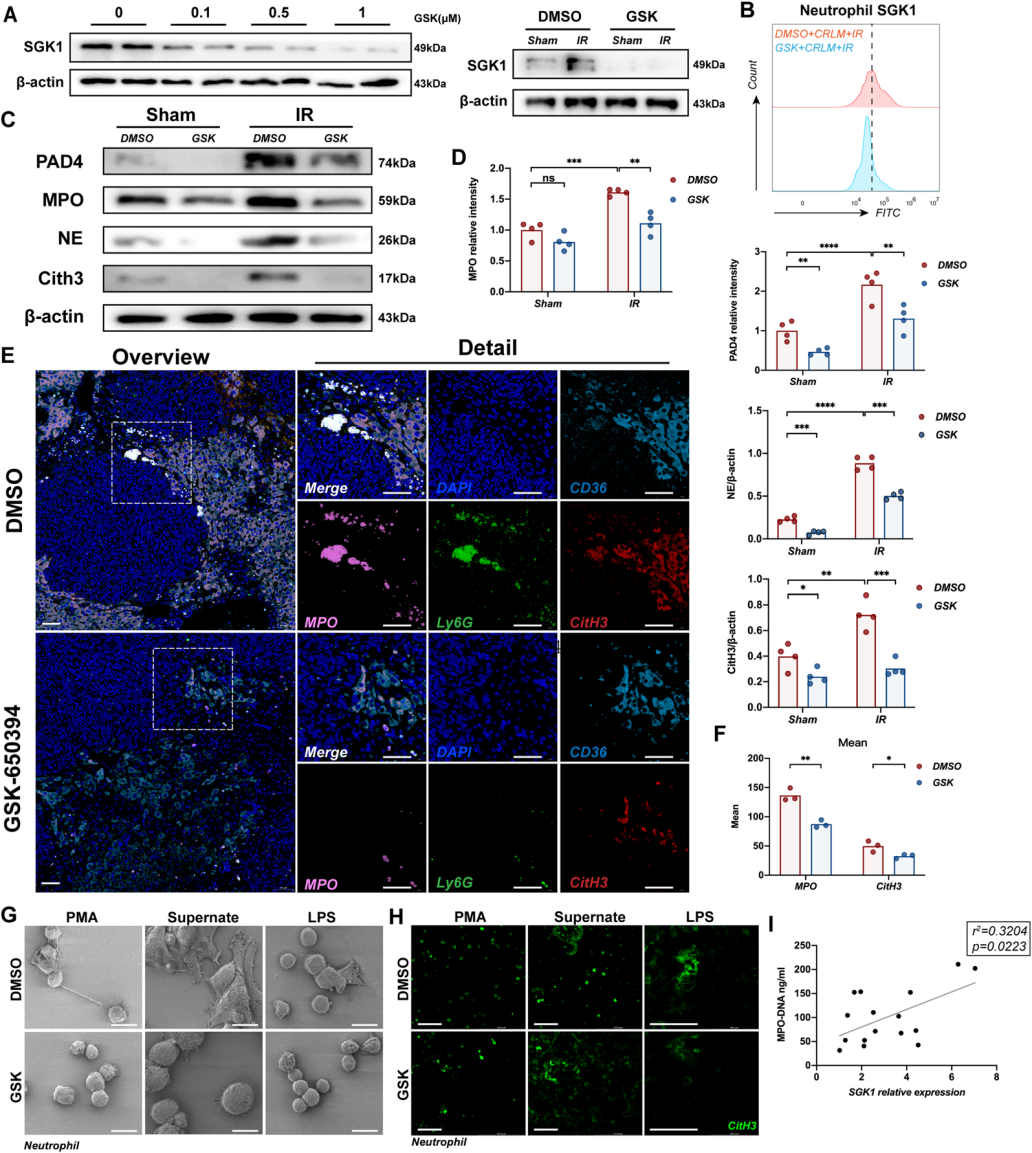
GSK-650394 regulates NETs formation in CRLM mice with IR background. Using GSK-650394 and then a 90-min warm ischemia model was constructed in mice, and the liver was removed after 6 h of reperfusion. **A** The expression of SGK1 was detected by western blot in ischemic liver or LO2. (n = 3 samples/group). **B** Before constructing CRLM-IR mouse model, GSK-650394 was injected intraperitoneally, and peripheral blood neutrophils were then isolated after 2 weeks of CRLM-IR surgery. The expression of SGK1 in circulating neutrophils of CRLM mice with IR background was detected by cytometry, and DMSO was injected intraperitoneally as control group (n = 3 samples/group). **C** The expression of MPO, NE, PAD4, and CitH3 was detected by western blot (n = 4 samples/group). **D** Quantification of MPO, NE, PAD4, CitH3 grayscale values (n = 4samples/group). **E** Immunofluorescence staining of CD36, MPO, Ly6G, CitH3 in livers of CRLM mouse undergoing IR (n = 4 samples/group). Scale bars, 50 μm. **F** Quantification of immunofluorescence (n = 4 samples/group). **G** The isolated neutrophils from circulation of wild type mice were pretreated with GSK-650394 or DMSO. In vitro, NETs were induced by PMA (200 nM), co-culture with MC38 cell line supernatant, and LPS (100 ng/ml). NETs were detected by scanning electron microscope (n = 4 samples/group). Scale bars, 10 μm. **H** Immunofluorescence staining of CitH3 (n = 4 samples/group), scale bars, 10 μm. **I** Correlation analysis of serum MPO-DNA (ng/ml) and relative mRNA expression in liver of patients with CRLM after surgery (n = 16 samples). All data represent the mean ± SD. *p < 0.05, **p < 0.01, ***p < 0.001****p < 0.0001

We have demonstrated that knockdown of SGK1 specifically targeting hepatocytes attenuates liver metastases exacerbated by hepatic IR. GSK-650394 is a competitive SGK1 inhibitor [55]. GSK-650394 has been validated in in vitro models to attenuate cerebral ischemia-reperfusion injury. [31–33] Related studies have shown that GSK-650394 exhibits antitumor activity in established breast cancer cell lines in vitro and in vivo [56]. Application of GSK-650394 to attenuates breast cancer lung metastasis in mice [57], and we wondered whether GSK-650394 had similar efficacy in CRLM. Surprisingly, intraperitoneal injection of GSK-650394 (MCE, China) before modeling resulted in the reduction of CRLM tumor burden in mice, compared with the group of mice with a specific knockout of hepatocytes SGK1 (Additional file 2: Fig. S6A, B). CRLM progression in the IR background was further delayed (Additional file 2: Fig. S6C, D), and the survival of mice was further prolonged (Additional file 2: Fig. S6E). We speculated that non-parenchymal cells other than hepatocytes expressing SGK1 contributed to the inflammatory progression of hepatic IR and IR-exacerbated tumor metastasis. The involvement of NETs in the regulation of hepatic IR has been generally demonstrated [24, 58], and is directly proportional to the progression of colorectal cancer liver metastases [13]. Recently, there have also been reports of the regulation of NETs by SGK1 [34]. We first examined the inhibitory efficiency of GSK-650394 in inhibiting SGK1 in vitro and in vivo (Fig. 5A), and the expression of SGK1 on neutrophils isolated from the peripheral blood of mice subjected to CRLM-IR was also inhibited detected by flow cytometry (Fig. 5B). The expression of NETs formation-related proteins was increased in the liver tissue and was reversed by GSK-650394 (Fig. 5C, D). Interestingly, we found that CitH3, MPO and ly6G co-expressed in the liver of CRLM mice undergoing IR, and the expressions of CitH3 and MPO were inhibited after using GSK-650394 (Fig. 5E, F). Furthermore, we detected lower levels of MPO-DNA in circulation of CRLM-IR mice using GSK-650394 (Fig. 5I). In vitro, we found that NETs induced by PMA inflammation, LPS inflammation, or tumor cell supernatant were all inhibited by GSK-650394 (Fig. 5G, H). The overexpression of Ly6G in the liver tissue of CRLM mice in the IR back-ground was also inhibited by GSK-650394 (Additional file 2: Fig. S8A, B).

Studies have shown that ERK activation is an important hub for NETs formation [59–61]. Besides, SGK1 regulates MEK/ERK signaling pathway in the regenerating liver [62]. Therefore, we speculate that GSK-650394 reduces NETs production by inhibiting ERK activation in neutrophils. We further constructed a mouse CRLM-IR model and found that ERK activation was inhibited by using GSK-650394 (Additional file 2: Fig. S7A, B), and with ERK inhibition, the generation of NETs was reduced in the IR model of mice or in vitro (Additional file 2: Fig. S7C, D).

### GSK-650394 inhibits the activation of PMN-MDSCs in CRLM Mice after IR

**Fig. 6:**
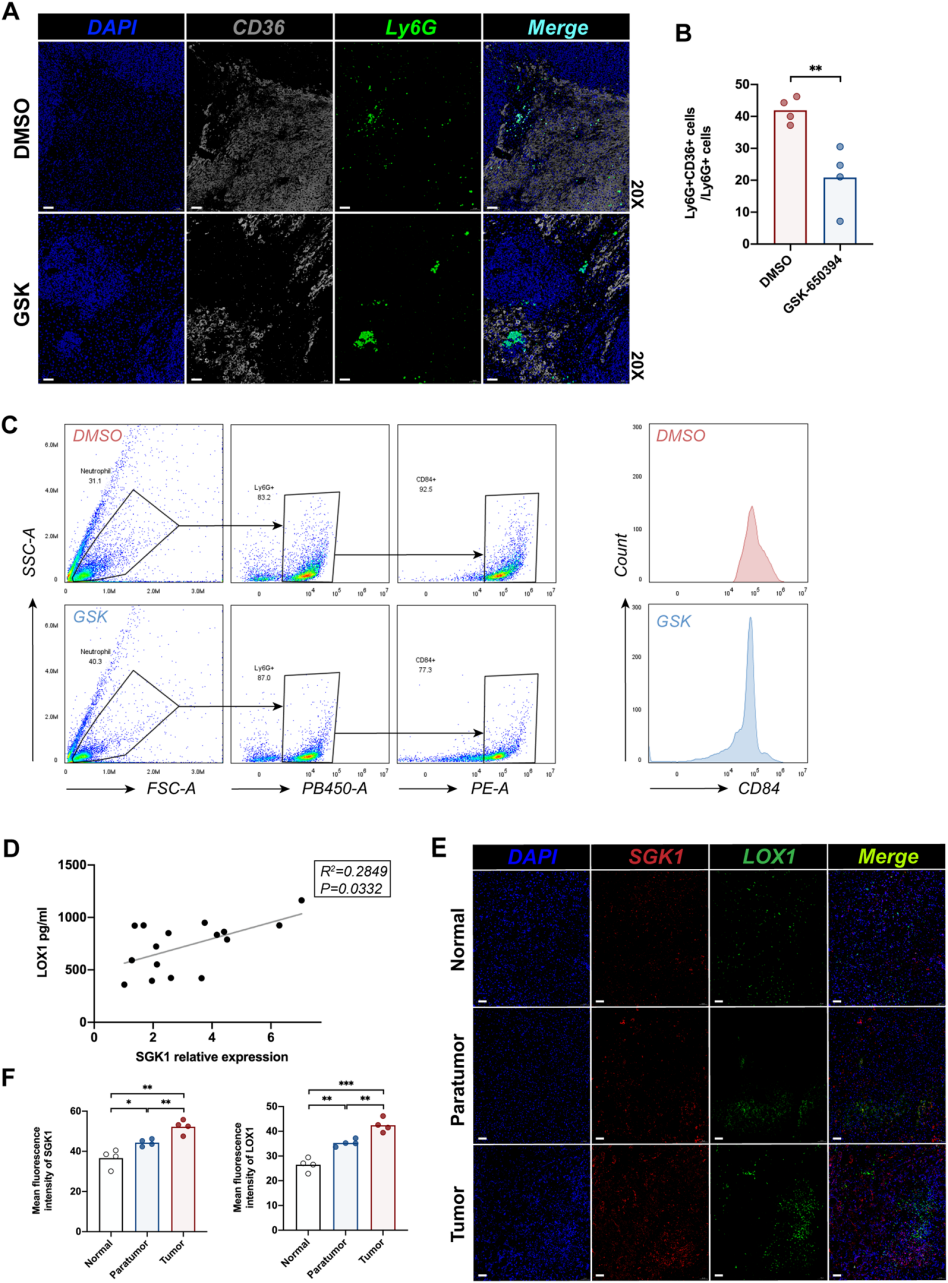
GSK-650394 inhibits the activation of PMN-MDSCs in CRLM Mice after IR. After intraperitoneal injection of GSK-650394, a mouse CRLM + IR model was established. **A** Immunofluorescence detection of Ly6G, CD36 expression in mouse liver (n = 4 samples/group). Scale bars, 50 μm. **B** Quantification of CD36 and Ly6G immunofluorescence, (n = 4 samples/group). **C** Peripheral blood neutrophils were extracted to detect CD84 expression by flow cytometry (n = 4 samples/group). **D** Elisa detection of LOX1 levels from serum from patients with CRLM 1 day after surgery. SGK1 expression of livers from patients with CRLM undergoing partial hepatectomy was detected by Quantitative RT-PCR. Correlation of LOX1 with SGK1 (n = 16 samples). **E** The expression of SGK1 and LOX1 in tumor and adjacent tumor of patients with CRLM was detected by immunofluorescence, and healthy liver tissue was used as control group (n = 4 samples/group). Scale bars, 50 μm. **F** Quantification of SGK1 and LOX1 immunofluorescence (n = 4 samples/group). All data represent the mean ± SD. *p < 0.05, **p < 0.01, ***p < 0.001****p < 0.0001

We found co-expression of CD36 with mpo and CitH3 during immunofluorescent staining of NETs (Fig. 5E). There is now ample evidence that lipid metabolism has been changed in MDSCs and that this plays a critical role in their differentiation and immunosuppressive functions [26, 63]. In the livers of mice treated with GSK-650394 and CRLM-IR surgery, the proportion of PMN-MDSCs decreased (Fig. 6A, B). CD84 has been identified as a robust MSDC-specific cell surface marker in breast cancers [64]. Strikingly, the expression of CD84 in peripheral blood neutrophils was downregulated in GSK-650394-treated mice (Fig. 6C).To further study how SGK1 regulates the involvement of non-parenchymal cells in liver metastasis, we collected 16 liver specimens from patients with CRLM. Recent review by the Gabrilovich group has shown lectin-type oxidized LDL receptor 1 (LOX1) has been noted as a specific marker of human PMN-MDSCs to distinguish these cells in blood or tumors in patients with cancer [26, 65, 66]. Serum LOX1 has been confirmed as a novel prognostic biomarker of colorectal cancer during recent research [67]. Therefore, we found a positive correlation between SGK1 expression in the liver and LOX1 levels from serum of these patients on the first postoperative day (Fig. 6D). While overexpressed SGK1 and LOX1 were found in the tumor and adjacent to the tumor in liver specimens from patients with CRLM ().

The expression of PMN-MDSC-related chemokines receptors such as CXCR2 and CXCR4 [68, 69], also decreased (Additional file 2: Fig. S1C).In vitro studies have also shown that GSK-650394 reduces the transcript levels of S100A8, S100A9, ARG1, and STAT3, which are capable of regulating immunosuppressive activity in neutrophils (Additional file 2: Fig. S9A). As expected, the production of PGE2 in the serum of CRLM mice after IR surgery was also inhibited by GSK-650394 (Additional file 2: Fig. S9B).

### Expression of peripheral SGK1 predicts postoperative prognosis in patients with CRLM

**Fig. 7:**
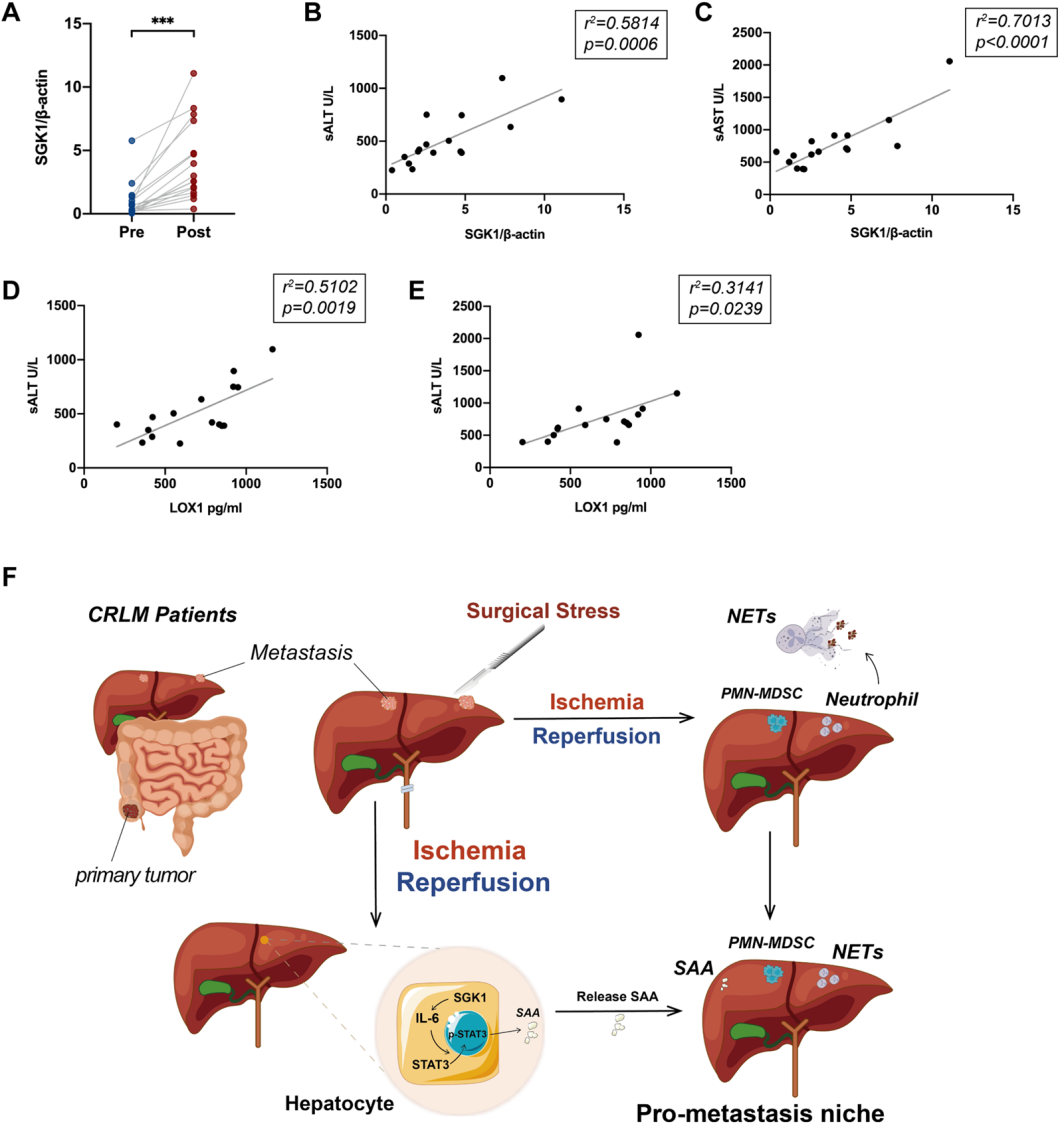
Expression of peripheral SGK1 predicts postoperative prognosis in patients with CRLM. Neutrophils were extracted from the peripheral blood of CRLM patients 1 day before or after surgery. **A** The expression of SGK1 mRNA in isolated neutrophils was detected by Quantitative RT-PCR 1 day before or after surgery (n = 16 samples). **B** and **C** Serum ALT and AST levels in CRLM patients 1 day after operation were detected by Elisa, and their correlation with SGK1 mRNA expression in peripheral blood neutrophils was analyzed (n = 16 samples). **D** and **E** As previously described, the level of LOX1 was detected in postoperative serum of CRLM patients, and analyzed its correlation with postoperative serum ALT and AST (n = 16 samples). **F** Schematic diagram of the signaling pathways involved in hepatic IR induced metastasis. Hepatic ischemia-reperfusion injury releases SAA by activating the SGK1/IL-6/STAT3 pathway, while inducing the infiltration of NETS and PMN-MDSC to form a pro-metastatic niche and promote postoperative recurrence of CRLM. All data represent the mean ± SD. *p < 0.05, **p < 0.01, ***p < 0.001****p < 0.0001

Peripheral blood samples were collected from 16 CRLM patients one day before surgery and one day after surgery. The mRNA levels of SGK1 in the serum neutrophils after surgery were higher than those before surgery (Fig. 7A). Surprisingly, we found that the mRNA levels of SKG1 in neutrophils in the peripheral blood of these patients were positively correlated with sALT and sAST levels one day after surgery (Fig. 7B, C). We also found that the levels of LOX1 in the serum of these patients were positively correlated with sALT and sAST levels (Fig. 7D, E). Our study showed that high expression of SGK1 or LOX1 in postoperative serum of patients probably predict poor prognosis. Taken together, our data indicates that hepatocyte SGK1 is a promising target for preventing IR-driven metastasis (Fig. 7F).

## Discussion

Colorectal cancer (CRC) is one of the most common malignancies. In addition, liver metastases in CRC patients are the most common cause of metastasis and the leading cause of death. Despite significant advances in diagnostic and therapeutic techniques, the survival rate of patients with CRLM remains low. As a complex cascade reaction process involving multiple factors and programs, CRLM involves complex and diverse molecular mechanisms [70]. Metastasis requires the circulation of tumor cells from the primary tumor site to distant organs that are “ready” to engraft disseminated tumor cells in a process known as pre-metastatic niche formation [26].

Recent studies have shown that hepatocytes form a pre-metastatic microenvironmental response to liver metastasis through the activation of IL-6/STAT3-triggered inflammation and further production of SAA accumulation, which is critical for liver metastasis, emphasizing the important role of hepatocytes in driving CRC liver metastasis [18]. In the canonical signaling pathway, IL-6 binds to the membrane-bound receptor IL-6R (mIL-6R) and then transmits the signal by recruiting and homodimerizing two glycoprotein 130 (gp130) subunits. This in turn activates the phosphorylation and activation of the JAK and STAT3 signaling pathways [71]. In our study, upregulated IL-6 in the context of IR in the mouse liver was inhibited by specific knockdown of SGK1 in hepatocytes, which further reduced the activation of STAT3. The accumulation of SAA was also reduced in the liver. The accumulation of SAA was also reduced in the liver. SAA, as previously confirmed, is associated with tumor metastasis [72–74].

We found that mice pretreated with GSK-650394 exhibited milder metastases after IR than mice with specifically knocked out hepatocyte SGK1. It has been shown that NETs are activated in response to hepatic IR and promote CRC liver metastasis [13]. Our study further demonstrates that GSK-650394 is involved in the generation of pre-metastatic niches in the colorectal cancer livers by inhibiting IR-infiltrating neutrophil SGK1/ERK/NETs signaling, thereby attenuating CRLM progression after IR. Regarding the mechanism of NETs promoting CRC metastasis, on the one hand, liver IR induces the formation of NETs, which in turn aggravates the damage of liver parenchyma cells [75]. It has also been shown that mouse neutrophil-derived NETs trigger HMGB1 release and activate TLR9-dependent pathways in cancer cells to promote their adhesion, proliferation, migration and invasion [13]. There are also studies suggesting that through mechanical trapping, the DNA grids of NETs trap circulating cancer cells, but are unable to kill or even harm these metastatic cells [76]. Neutrophil extracellular traps (NETs) consist of chromatin DNA filaments coated with granulin that are released by neutrophils to capture microorganisms. Tumor cells sense extracellular DNA through the membrane protein CCDC25 for capture by NETs [25]. β1 integrin expression in NETs and cancer cells is important for the adhesion of circulating cancer cells to NETs [77].

In addition, we also found that the PMN-MDSC-related serum marker LOX1 was positively correlated with SGK1 in the postoperative serum of patients with CRLM. The number of PMN-MDSCs is significantly increased in the tumors of HNC, colon cancer, and NSCLC patients, and the combination of serum LOX1 with neutrophil markers allows the detection of PMN-MDSC in tissues [65]. Another study used LOX1 as a serum marker for CRC prognosis [67]. We further verified this in the mouse CRLM + IR model, and the peripheral serum CD84 level was positively correlated with the SGK1 level after two weeks of CRLM + IR in mice. GSK-650394 inhibited the level of peripheral CD84 in mice. CD84 is a cell surface receptor of the signaling lymphocytic activation molecule (SLAM) family expressed on some immune cell types and has been identified as a surface marker for improved detection and enrichment of MDSCs in breast cancers [64]. We used CD36 to delabel MDSCs in mouse liver tissue. CD36 was recently identified as a marker of tumor-infiltrating PMN-MDSCs, and it plays an immunosuppressive role in mediating STAT3 signaling in MDSCs [63]. The results showed that GSK-650394 effectively inhibited the IR background CD36 expression of infiltrated Ly6G cells in the livers of CRLM mice, indicating that GSK-650394 also inhibited the peripheral recruitment of PMN-MDSCs and their infiltration into the liver. PMN-MDSCs in the pre-metastatic niche may facilitate tumor cell escape by suppressing immune cells, inducing matrix remodeling, and promoting angiogenesis, which in turn promotes tumor cell engraftment [28].

## Conclusions

In summary, our study demonstrated that activation of IL-6/STAT3/SAA signaling by hepatocyte SGK1 in response to IR promotes the formation of a pre-metastatic niche in the liver of CRC, and that tumor-associated NETs and PMN-MDSCs participate in the formation of CRC in an SGK1-dependent manner. Liver metastases anterior niche. As surgery remains the appropriate and only potential treatment for patients with metastatic disease, we aimed to better understand the mechanisms by which surgery promotes tumor recurrence. Treatment strategies to prevent tumor recurrence after surgery should be explored. Therefore, targeting SGK1 is a therapeutic strategy. Our findings provide preliminary evidence that the preoperative inhibition of SGK1 by administration can significantly attenuate IR injury in mice and the progression of CRLM after IR, potentially paving the way for future clinical trials.

## Supplementary Information

**Additional file 1.** Supplementary Figure 1–9.

**Additional file 2: Figure S1. **Gal-siSGK1 reverses the activation of inflammatory factors in liver after IR. Livers of wild type mice were first subjected to 70% warm ischemia, followed by reperfusion for 6h. (A) qRT-PCR analysis of IL-1β, TNF-α, INOS, CXCL2, CXCL10 and IL-10 in ischemic livers (n=6samples/group). (B) Serum IL-1β level was detected by Elisa (n=4samples/group). (C) qRT-PCR analysis of CXCR2, CXCR4 in ischemic livers (n=6samples/group). All data represent the mean ± SD. *p < 0.05, **p < 0.01, ***p < 0.001, ****p < 0.0001. **Figure S2. **Gal-siSGK1 alleviates apoptosis in vitro cell lines after H/R. (A) Using Gal-siSGK1, and then the apoptosis of AML cell line after H/R were detected by flow cytometry (n=3samples/group). (B) The apoptosis of LO2 cell line after H/R were detected by flow cytometry (n=3samples/group). All data represent the mean ± SD. *p < 0.05, **p < 0.01, ***p < 0.001, ****p < 0.0001. **Figure S3. **STAT3 inhibitor alleviates CRLM after IR. Before constructing the mouse IR model, STAT3 inhibitor was injected intraperitoneally. (A) The efficiency of STAT3 knockout was detected by WB (n=3samples/group). (B) Detection of mouse liver function by examining serum ALT and AST (n=4samples/group). (C) HE staining to detect liver tissue damage (n=3samples/group). Scale bars, 50μm. (D) Tunel staining to detect hepatocyte apoptosis (n=4samples/group). Scale bars, 50μm. (E) Quantification of Tunel staining (n=4samples/group). Gal-siSGK1 and GSK-650394 were used to knock down mouse SGK1, respectively, and then a mouse CRLM+IR model was constructed. (F) Representative images of tumor (n=4samples/group). (G) HE staining of mouse liver. (n=3samples) Scale bars, 500μm. (H) Liver surface metastases number of stoves (n=4samples). (I) Quantification of tumor area (n=4samples/group). All data represent the mean ± SD. *p < 0.05, **p < 0.01, ***p < 0.001, ****p < 0.0001. **Figure S4. **Overexpression of SAA in patients with CRLM depends on SGK1. Liver specimens from CRLM patients and peripheral blood 1 day after surgery were collected. (A) Immunohistochemistry of liver SAA paratumor tissue, healthy liver tissue as control group (n=4samples/group). (B) Immunohistochemical quantification (n=4samples/group). (C) Serum SAA level from patients with CRLM was examined 1 day after surgery by Elisa and the correlation analysis of liver SGK1 relative expression with serum SAA levels (n=16samples). All data represent the mean ± SD. *p < 0.05, **p < 0.01, ***p < 0.001, ****p < 0.0001. **Figure S5. **Liver metastasis regulated by hepatocyte SGK1 is independent of NRF2 crosstalk. Using Gal-siSGK1 or Gal-siCtrl and then a mouse IR model was constructed. (A) Expression of NRF2 in hepatocytes was detected by western blot (n=3samples/group). (B) qRT-PCR analysis of NRF2 in hepatocytes isolated from ischemic livers (n=4samples/group). (C) Immunofluorescence staining of SGK1, NRF2 and HNF-4α in ischemic livers or control livers, scale bars, 50μm (n=4samples/group). (D) Immunohistochemistry staining of NRF2 in ischemic livers or sham livers, scale bars, 20μm. (n=4samples/group). (E) Gal-siSGK1 or Gal-siCtrl were used to knock down SGK1 in hepatocytes，and then a mouse CRLM+IR model was constructed. Immunofluorescence staining of SGK1, NRF2 and HNF-4α, scale bars, 200μm (n=4samples/group). (F) Immunohistochemistry staining of NRF2 in CRLM livers, scale bars, 50μm. (n=4samples/group). All data represent the mean ± SD. *p < 0.05, **p < 0.01, ***p < 0.001****p < 0.0001. **Figure S6. **Administration of GSK-650394 attenuates liver IR injury and CRLM progression in mice compared to specific knockout of hepatocyte SGK1. Gal-siSGK1 and GSK-650394 were used to knock down mouse SGK1, respectively, and then a mouse CRLM+IR model was constructed. (A) Physical pictures of the tumor (n=6samples/group). (B) Number of metastases on liver surface (n = 6samples/group). (C and D) Representative images of bioluminescence and statistics were shown (n = 4samples/group). (E) Survival (n = 8samples/group). All data represent the mean ± SD. *p < 0.05, **p < 0.01, ***p < 0.001, ****p < 0.0001. **Figure S7. **SGK1 induces the generation of NETs by activating ERK. Mouse neutrophils were isolated in vitro and placed in a six-well plate first. Then GSK-650394 was added. To induce NETs, LPS (100ng/ml), PMA (200nM) and co-culture with tumor cell supernatants were used. (A) Expression of ERK1/2, p-ERK1/2 was detected by western blot (n=3samples/group). (B) Quantification of p-ERK/ERK. (n=3samples/group). (C) MEK inhibitor (U0126, MCE, China) was used to block ERK. NETs were then induced as mentioned before and were detected by scanning electron microscopy. Scale bars, 10μm. (n=4samples/group). (D) Expression of CitH3 in CRLM-IR mice liver treated with U0126 in vivo was detected by western blot (n=3samples/group). All data represent the mean ± SD. *p < 0.05, **p < 0.01, ***p < 0.001, ****p < 0.0001. **Figure S8: **Recruitment of Neutrophils in mice undergoing CRLM+IR is blocked by GSK-650394. GSK-650394 was injected intraperitoneally into mice to construct a mouse CRLM+IR model, and the mouse livers were harvested two weeks later. (A) Immunohistochemical detection of ly6G, mice injected intraperitoneally with DMSO as control group, (n=3samples/group). Scale bars, 50μm. (B) Immunohistochemical quantification (n=3samples/group). All data represent the mean ± SD. *p < 0.05, **p < 0.01, ***p < 0.001, ****p < 0.0001. **Figure S9: **GSK-650394 inhibits key biochemical signatures of PMN-MDSCs. GSK-650394 was injected intraperitoneally into mice to construct a mouse CRLM+IR model. The livers and the serum were harvested two weeks later. (A) The mRNA level of S100A8、S100A9、ARG1、STAT3 in livers were detected by qPCR (n=4samples/group). (B) The expression of serum PGE2 and were detected by Elisa (n=4samples/group). All data represent the mean ± SD. *p < 0.05, **p < 0.01, ***p < 0.001, ****p < 0.0001.

## Data Availability

Data available on request from the authors.The data that support the findings of this study are available from the corresponding author upon reasonable request.

## Abbreviations

CRLM: Colorectal liver metastasis
DAMP: Danger-associated molecular patterns
ERK: Extracellular signal-regulated kinase
iNOS: Inducible nitric-oxide synthase
IR: Ischemia reperfusion
MDSC: Myeloid-derived suppressor cell
MPO: Myeloperoxidase
NETs: Neutrophil extracellular trap
NRF2: Nuclear factor E2-related factor 2
PAMP: Pathogen-associated molecular pattern
PMN: Polymorphonuclear cells
PMN-MDSC: Polymorphonuclear MDSC
ROS: Reactive oxygen species
SAA: Serum amyloid A
sALT: Serum alanine aminotransferase
sAST: Serum aspartate aminotransferase
SGK1: Serum/glucocorticoid regulated kinase 1
STAT3: Signal transducer and activator of transcription 3
TUNEL: Terminal deoxynucleotidyl transferase-mediated deoxyuridine triphosphate nick-end labeling
