# Supplementary material for: Hepatocyte SGK1 activated by hepatic ischemia-reperfusion promotes the recurrence of liver metastasis via IL-6/STAT3
Source: J Transl Med. 2023 Feb 14;21:121. doi: 10.1186/s12967-023-03977-z (PMC9926712; doi:10.1186/s12967-023-03977-z)
Supplement: Supplementary file 2 — Additional file 2: Figure S1. Gal-siSGK1 reverses the activation of inflammatory factors in liver after IR. Livers of wild type mice were first subjected to 70% warm ischemia, followed by reperfusion for 6h. (A) qRT-PCR analysis of IL-1β, TNF-α, INOS, CXCL2, CXCL10 and IL-10 in ischemic livers (n=6samples/group). (B) Serum IL-1β level was detected by Elisa (n=4samples/group). (C) qRT-PCR analysis of CXCR2, CXCR4 in ischemic livers (n=6samples/group). All data represent the mean ± SD. *p < 0.05, **p < 0.01, ***p < 0.001, ****p < 0.0001. Figure S2. Gal-siSGK1 alleviates apoptosis in vitro cell lines after H/R. (A) Using Gal-siSGK1, and then the apoptosis of AML cell line after H/R were detected by flow cytometry (n=3samples/group). (B) The apoptosis of LO2 cell line after H/R were detected by flow cytometry (n=3samples/group). All data represent the mean ± SD. *p < 0.05, **p < 0.01, ***p < 0.001, ****p < 0.0001. Figure S3. STAT3 inhibitor alleviates CRLM after IR. Before constructing the mouse IR model, STAT3 inhibitor was injected intraperitoneally. (A) The efficiency of STAT3 knockout was detected by WB (n=3samples/group). (B) Detection of mouse liver function by examining serum ALT and AST (n=4samples/group). (C) HE staining to detect liver tissue damage (n=3samples/group). Scale bars, 50μm. (D) Tunel staining to detect hepatocyte apoptosis (n=4samples/group). Scale bars, 50μm. (E) Quantification of Tunel staining (n=4samples/group). Gal-siSGK1 and GSK-650394 were used to knock down mouse SGK1, respectively, and then a mouse CRLM+IR model was constructed. (F) Representative images of tumor (n=4samples/group). (G) HE staining of mouse liver. (n=3samples) Scale bars, 500μm. (H) Liver surface metastases number of stoves (n=4samples). (I) Quantification of tumor area (n=4samples/group). All data represent the mean ± SD. *p < 0.05, **p < 0.01, ***p < 0.001, ****p < 0.0001. Figure S4. Overexpression of SAA in patients with CRLM depends on SGK1. Liver specimens from CRLM [file 12967_2023_3977_MOESM2_ESM.docx]

***Additional file Doc***

**Hepatocyte SGK1 activated by hepatic ischemia-reperfusion promotes the recurrence of liver metastasis via IL-6/STAT3**

Xiangdong Li1,a,b,c, Ziyi Wang1,a,b,c, Chenyu Jiao1,a,b,c, Yu Zhanga,b,c, Nan Xiaa,b,c, Wenjie Yua,b,c, Xuejiao Chene, Likalamu Pascalia Wikanaa,b,c, Yue Liue, Linfeng Suna,b,c, Minhao Chena,b,c, Yuhao Xiaoa,b,c, Yuhua Shi#,d, Sheng Han #,a,b,c, Liyong Pu#,a,b,c

**Table of contents**

Fig. S1 ...............................................................................................................................................2

Fig. S2 ...............................................................................................................................................3

Fig. S3 ...............................................................................................................................................4

Fig. S4 ...............................................................................................................................................4

Fig. S5 ...............................................................................................................................................5

Fig. S6 ...............................................................................................................................................6

Fig. S7 ...............................................................................................................................................7

Fig. S8 ...............................................................................................................................................8

Fig. S9 ...............................................................................................................................................9

**
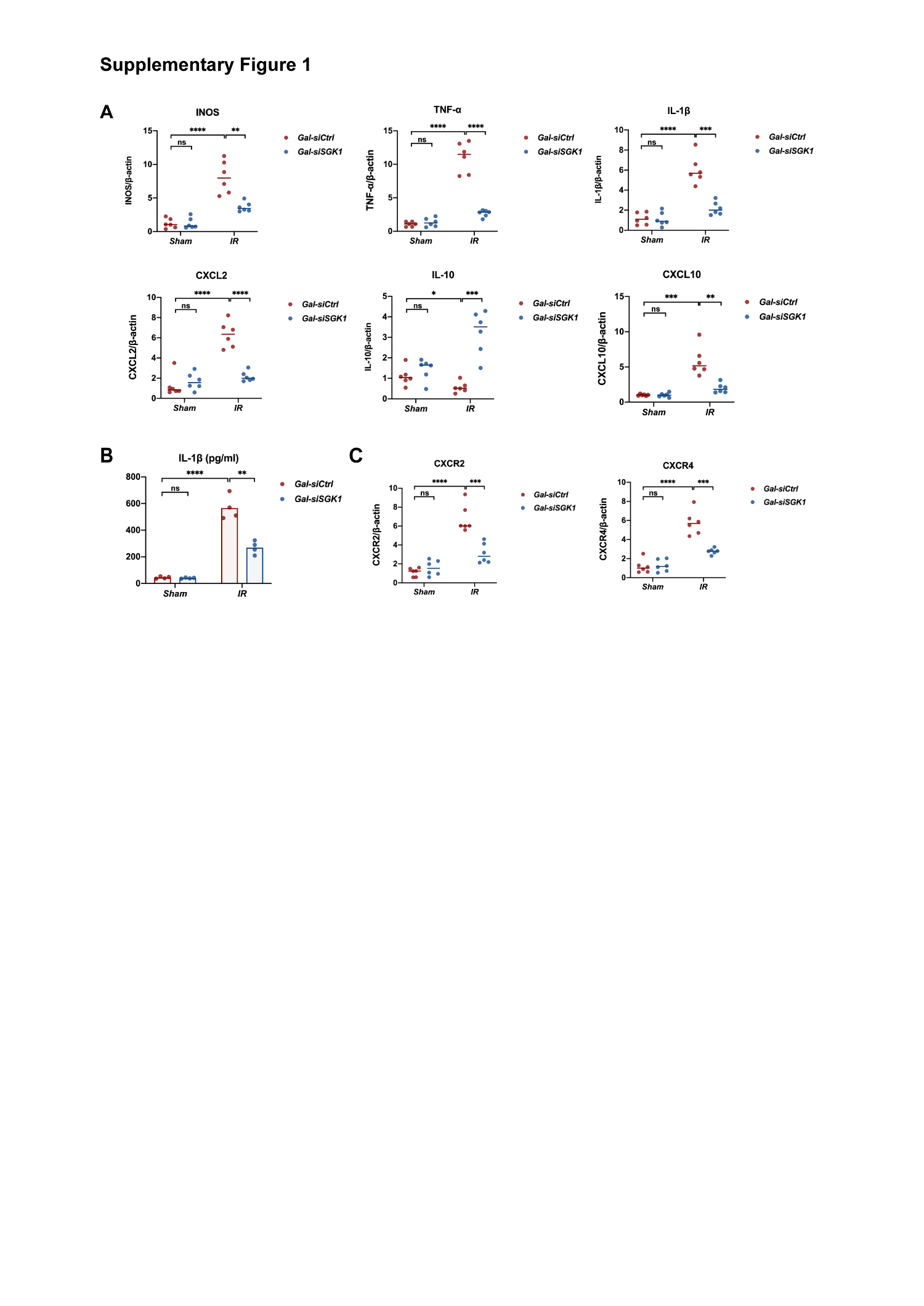
**

***FigureS1: Gal-siSGK1 reverses the activation of inflammatory factors in liver after IR***

Livers of wild type mice were first subjected to 70% warm ischemia, followed by reperfusion for 6h. (A) qRT-PCR analysis of IL-1β, TNF-α, INOS, CXCL2, CXCL10 and IL-10 in ischemic livers (n=6samples/group). (B) Serum IL-1β level was detected by Elisa (n=4samples/group). (C) qRT-PCR analysis of CXCR2, CXCR4 in ischemic livers (n=6samples/group). All data represent the mean ± SD. *p < 0.05, **p < 0.01, ***p < 0.001, ****p < 0.0001.


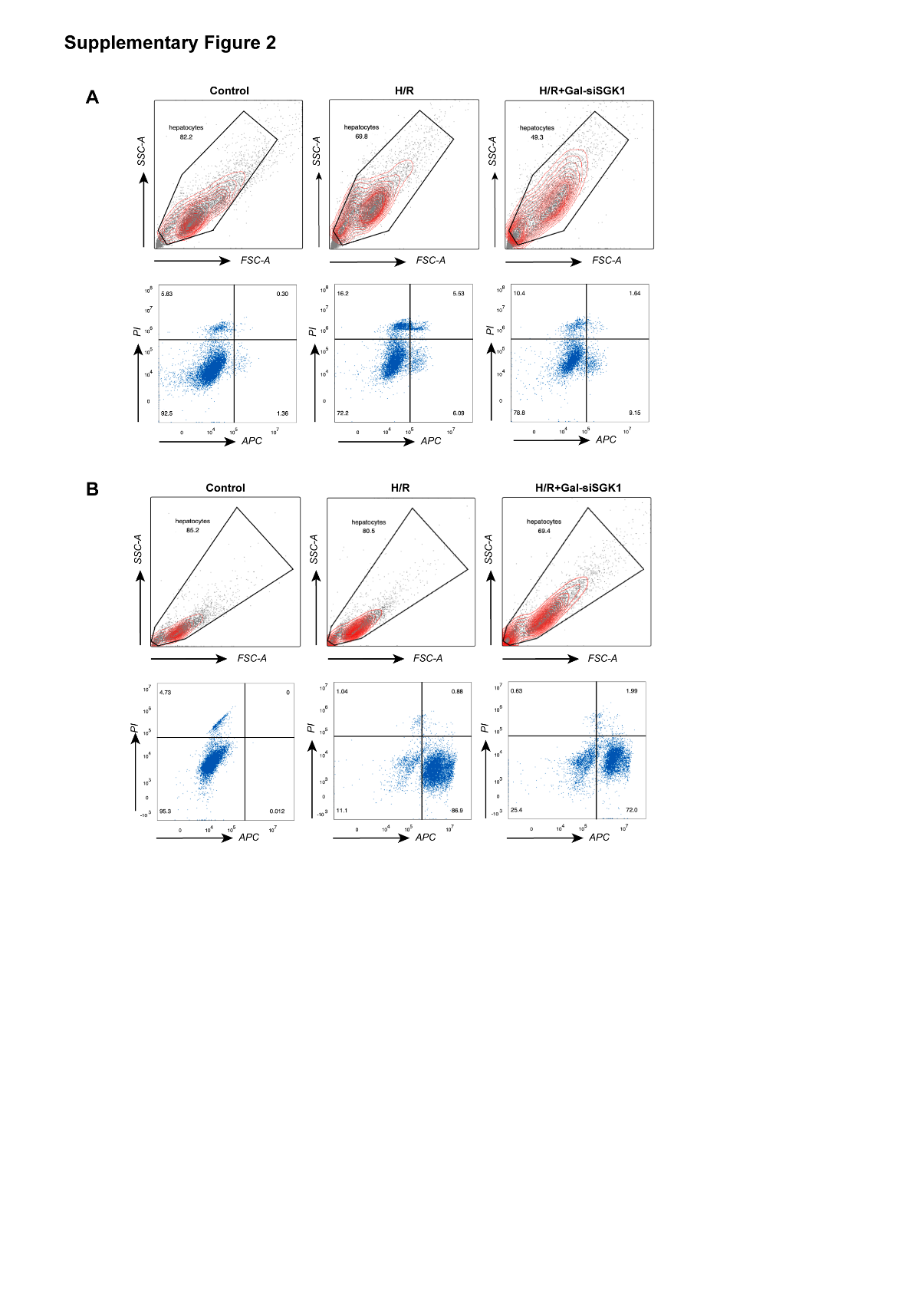


***Figure S2: Gal-siSGK1 alleviates apoptosis in vitro cell lines after H/R***

(A) Using Gal-siSGK1, and then the apoptosis of AML cell line after H/R were detected by flow cytometry (n=3samples/group). (B) The apoptosis of LO2 cell line after H/R were detected by flow cytometry (n=3samples/group). All data represent the mean ± SD. *p < 0.05, **p < 0.01, ***p < 0.001, ****p < 0.0001.


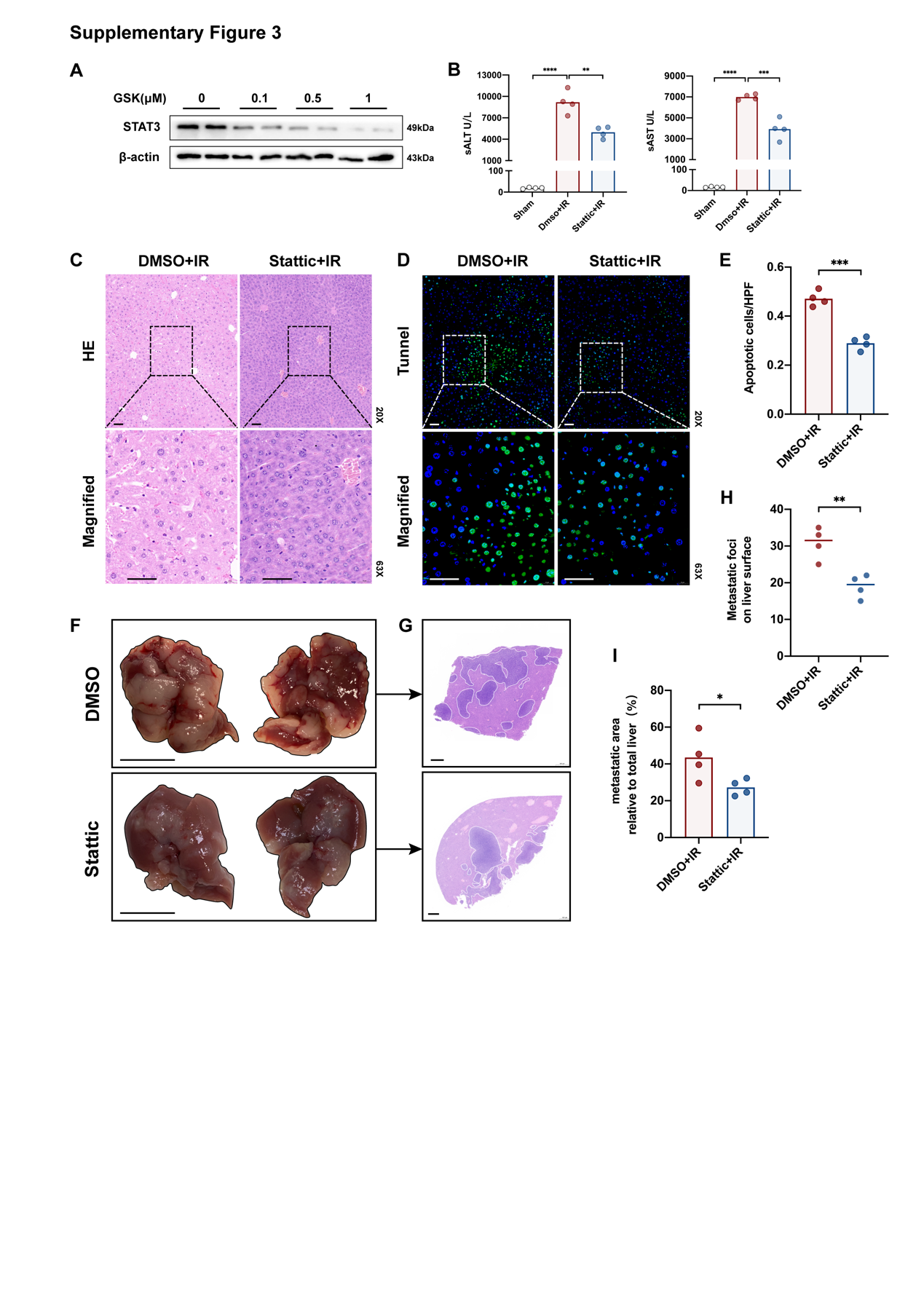


***Figure S3: STAT3 inhibitor alleviates CRLM after IR***

Before constructing the mouse IR model, STAT3 inhibitor was injected intraperitoneally. (A) The efficiency of STAT3 knockout was detected by WB (n=3samples/group). (B) Detection of mouse liver function by examining serum ALT and AST (n=4samples/group). (C) HE staining to detect liver tissue damage (n=3samples/group). Scale bars, 50μm. (D) Tunel staining to detect hepatocyte apoptosis (n=4samples/group). Scale bars, 50μm. (E) Quantification of Tunel staining (n=4samples/group). Gal-siSGK1 and GSK-650394 were used to knock down mouse SGK1, respectively, and then a mouse CRLM+IR model was constructed. (F) Representative images of tumor (n=4samples/group). (G) HE staining of mouse liver. (n=3samples) Scale bars, 500μm. (H) Liver surface metastases number of stoves (n=4samples). (I) Quantification of tumor area (n=4samples/group). All data represent the mean ± SD. *p < 0.05, **p < 0.01, ***p < 0.001, ****p < 0.0001.


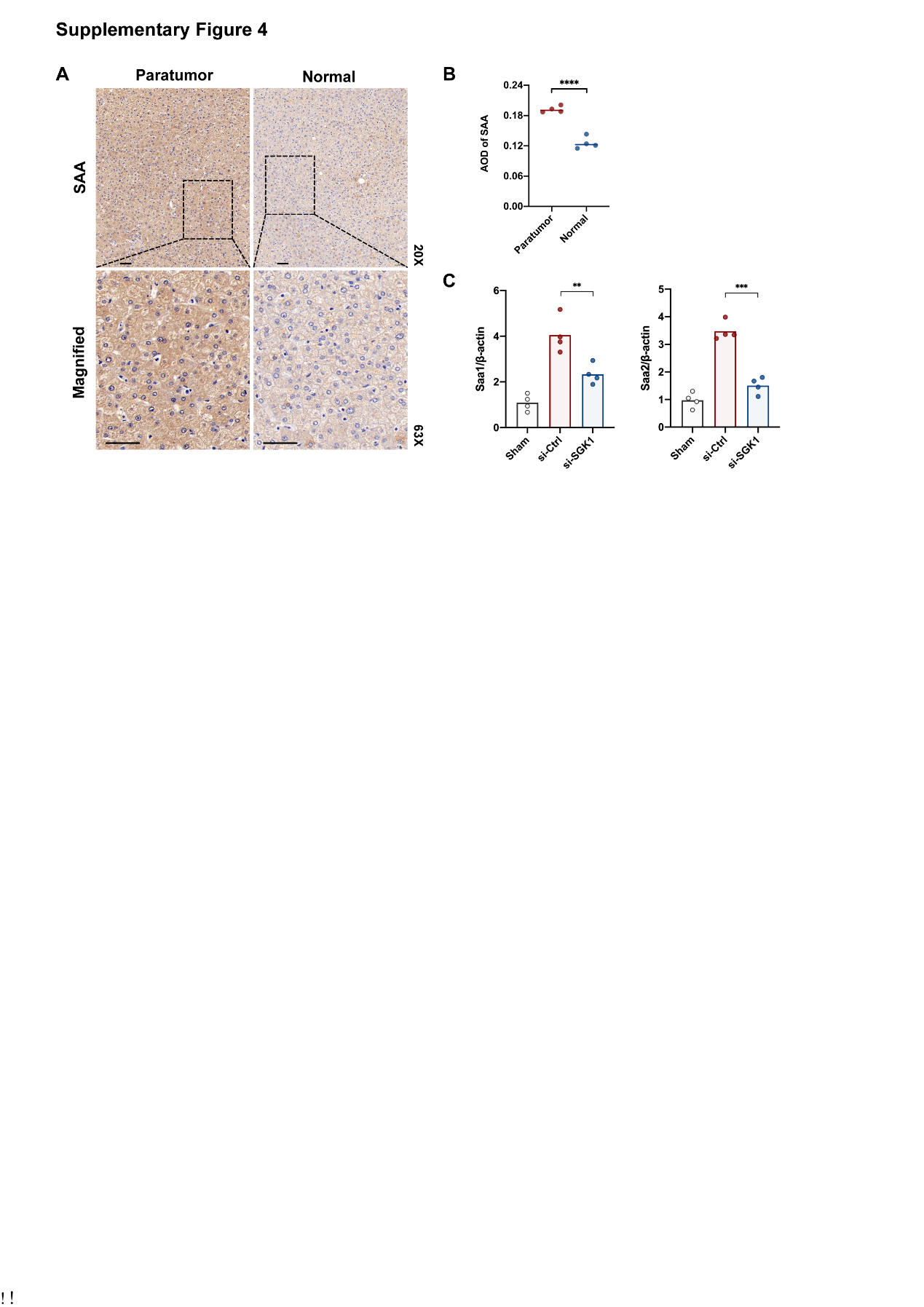


***Figure S4: Overexpression of SAA in patients with CRLM depends on SGK1***

Liver specimens from CRLM patients and peripheral blood 1 day after surgery were collected. (A) Immunohistochemistry of liver SAA paratumor tissue, healthy liver tissue as control group (n=4samples/group). (B) Immunohistochemical quantification (n=4samples/group). (C) Serum SAA level from patients with CRLM was examined 1 day after surgery by Elisa and the correlation analysis of liver SGK1 relative expression with serum SAA levels (n=16samples). All data represent the mean ± SD. *p < 0.05, **p < 0.01, ***p < 0.001, ****p < 0.0001.


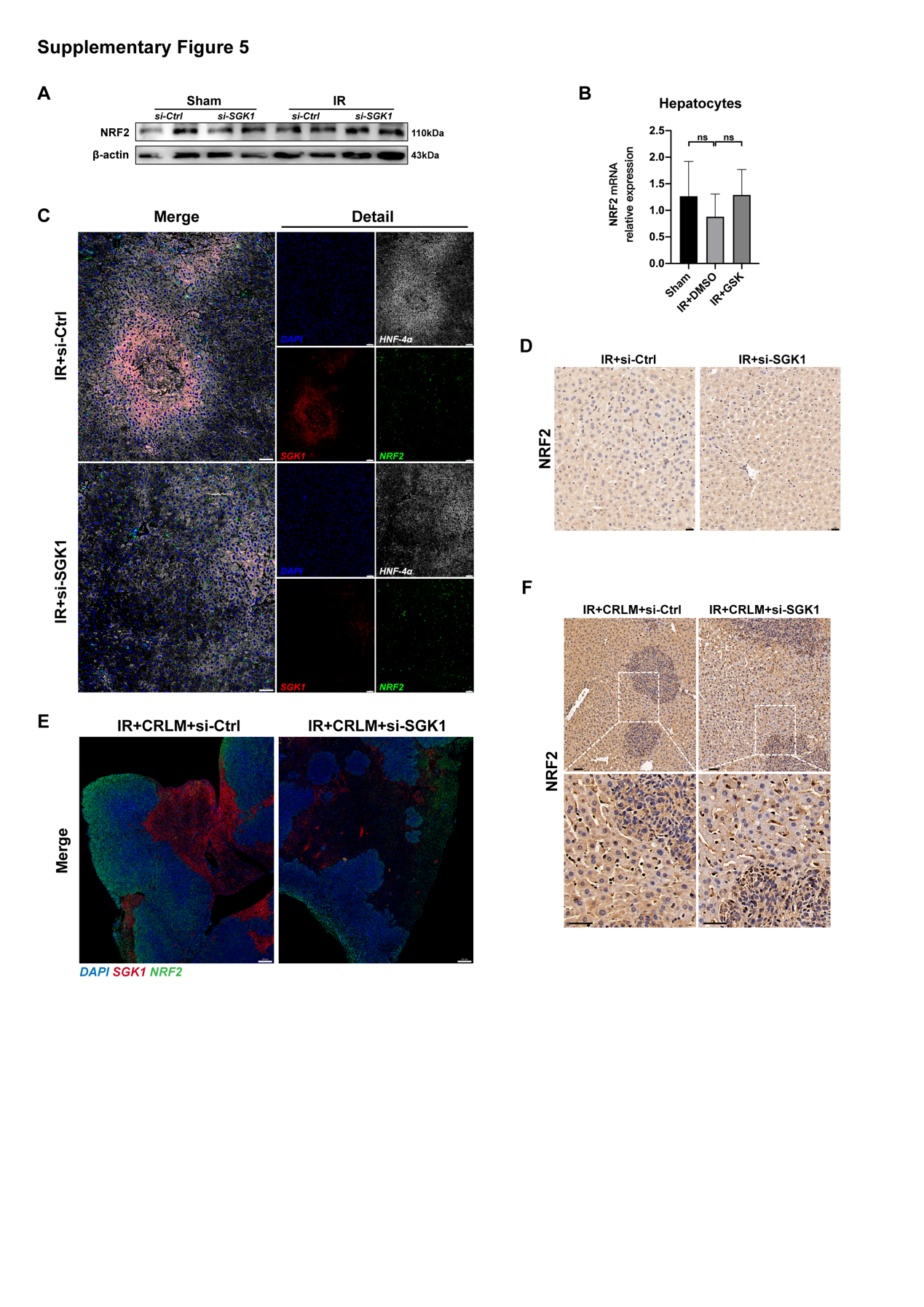


***Figure S5: Liver metastasis regulated by hepatocyte SGK1 is independent of NRF2 crosstalk***

Using Gal-siSGK1 or Gal-siCtrl and then a mouse IR model was constructed. (A) Expression of NRF2 in hepatocytes was detected by western blot (n=3samples/group). (B) qRT-PCR analysis of NRF2 in hepatocytes isolated from ischemic livers (n=4samples/group). (C) Immunofluorescence staining of SGK1, NRF2 and HNF-4α in ischemic livers or control livers, scale bars, 50μm (n=4samples/group). (D) Immunohistochemistry staining of NRF2 in ischemic livers or sham livers, scale bars, 20μm. (n=4samples/group). (E) Gal-siSGK1 or Gal-siCtrl were used to knock down SGK1 in hepatocytes，and then a mouse CRLM+IR model was constructed. Immunofluorescence staining of SGK1, NRF2 and HNF-4α, scale bars, 200μm (n=4samples/group). (F) Immunohistochemistry staining of NRF2 in CRLM livers, scale bars, 50μm. (n=4samples/group). All data represent the mean ± SD. *p < 0.05, **p < 0.01, ***p < 0.001****p < 0.0001.


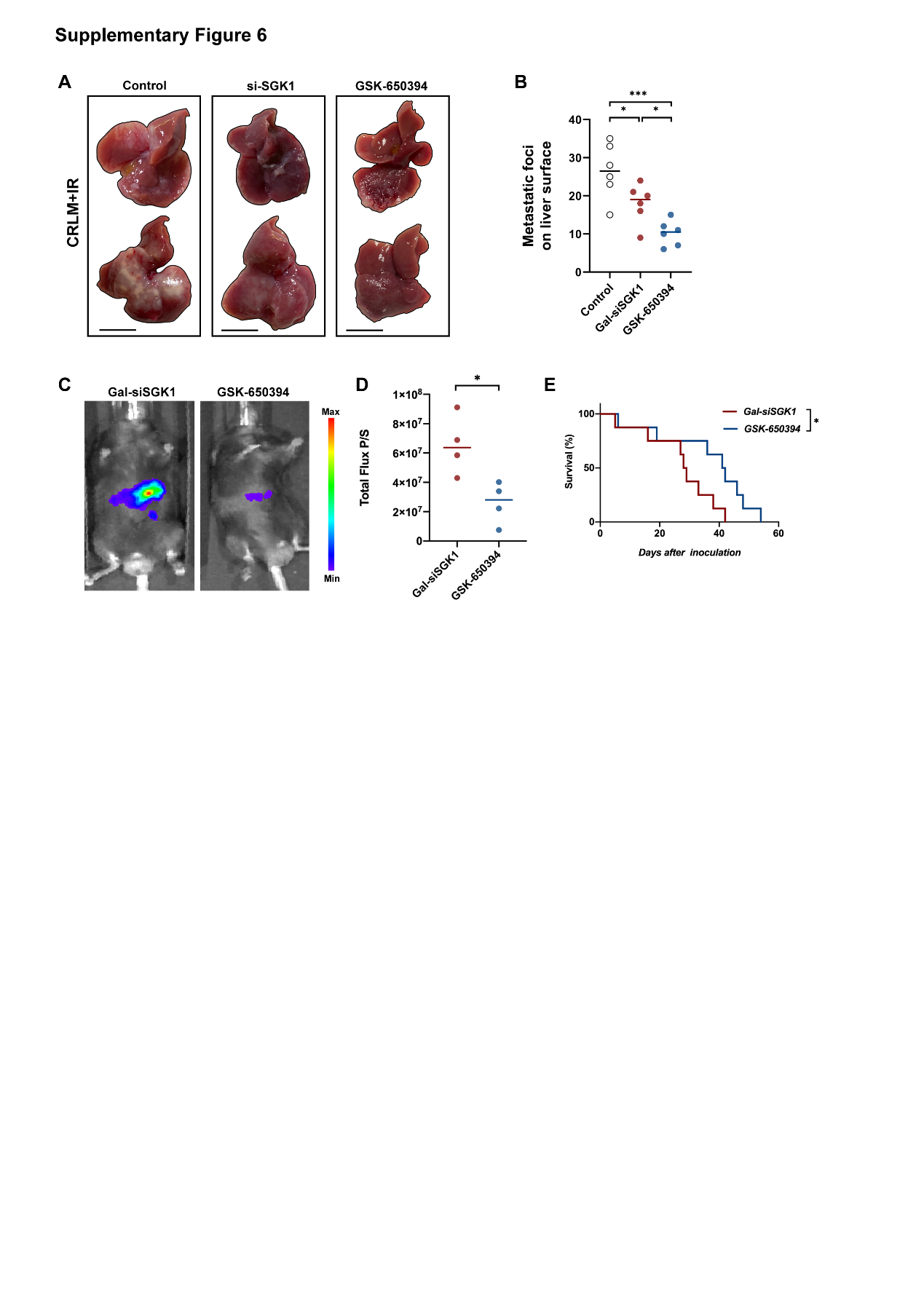


***Figure S6: Administration of GSK-650394 attenuates liver IR injury and CRLM progression in mice compared to specific knockout of hepatocyte SGK1***

Gal-siSGK1 and GSK-650394 were used to knock down mouse SGK1, respectively, and then a mouse CRLM+IR model was constructed. (A) Physical pictures of the tumor (n=6samples/group). (B) Number of metastases on liver surface (n = 6samples/group). (C and D) Representative images of bioluminescence and statistics were shown (n = 4samples/group). (E) Survival (n = 8samples/group). All data represent the mean ± SD. *p < 0.05, **p < 0.01, ***p < 0.001, ****p < 0.0001.


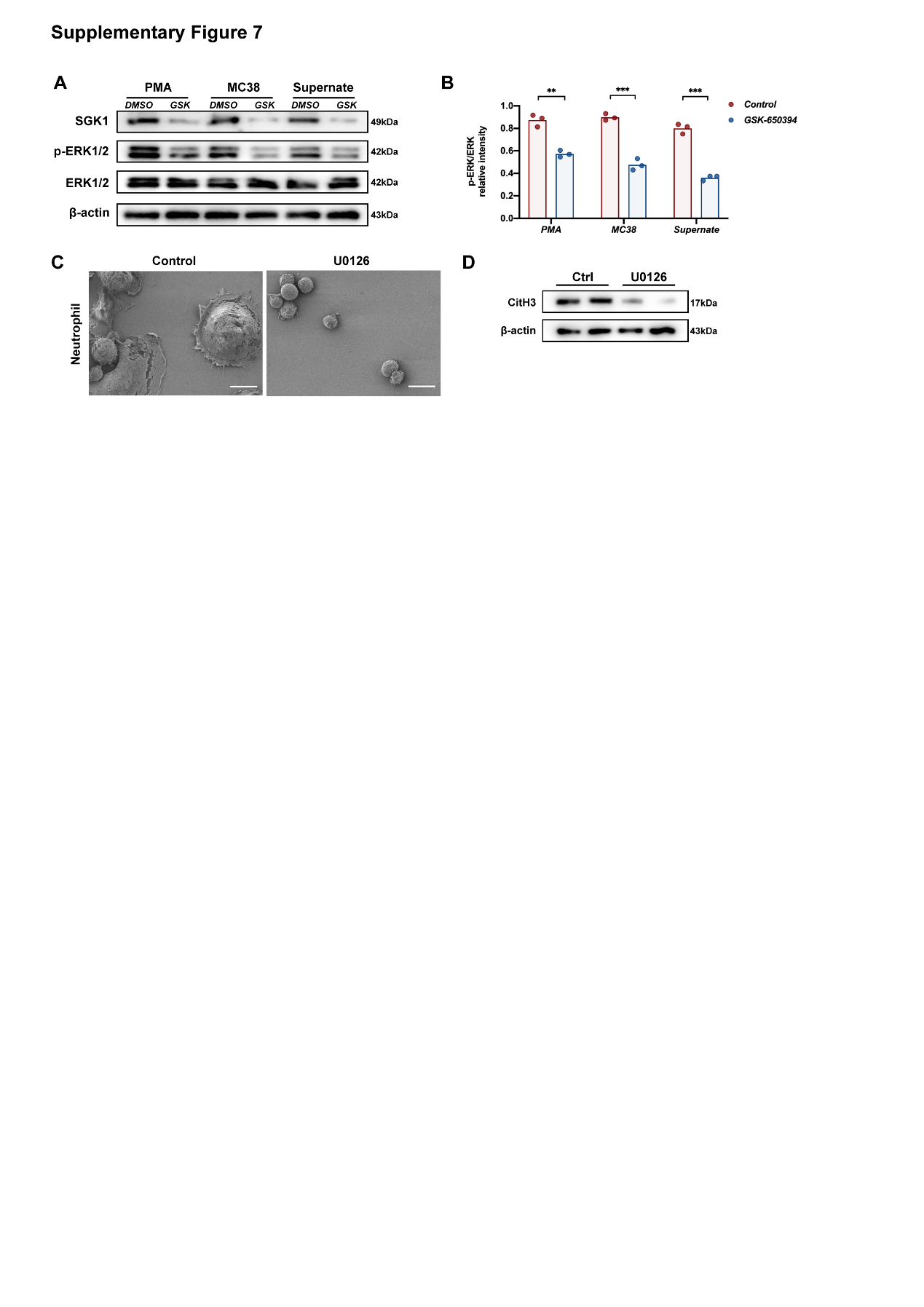


***Figure S7: SGK1 induces the generation of NETs by activating ERK***

Mouse neutrophils were isolated in vitro and placed in a six-well plate first. Then GSK-650394 was added. To induce NETs, LPS (100ng/ml), PMA (200nM) and co-culture with tumor cell supernatants were used. (A) Expression of ERK1/2, p-ERK1/2 was detected by western blot (n=3samples/group). (B) Quantification of p-ERK/ERK. (n=3samples/group). (C) MEK inhibitor (U0126, MCE, China) was used to block ERK. NETs were then induced as mentioned before and were detected by scanning electron microscopy. Scale bars, 10μm. (n=4samples/group). (D) Expression of CitH3 in CRLM-IR mice liver treated with U0126 in vivo was detected by western blot (n=3samples/group). All data represent the mean ± SD. *p < 0.05, **p < 0.01, ***p < 0.001, ****p < 0.0001.


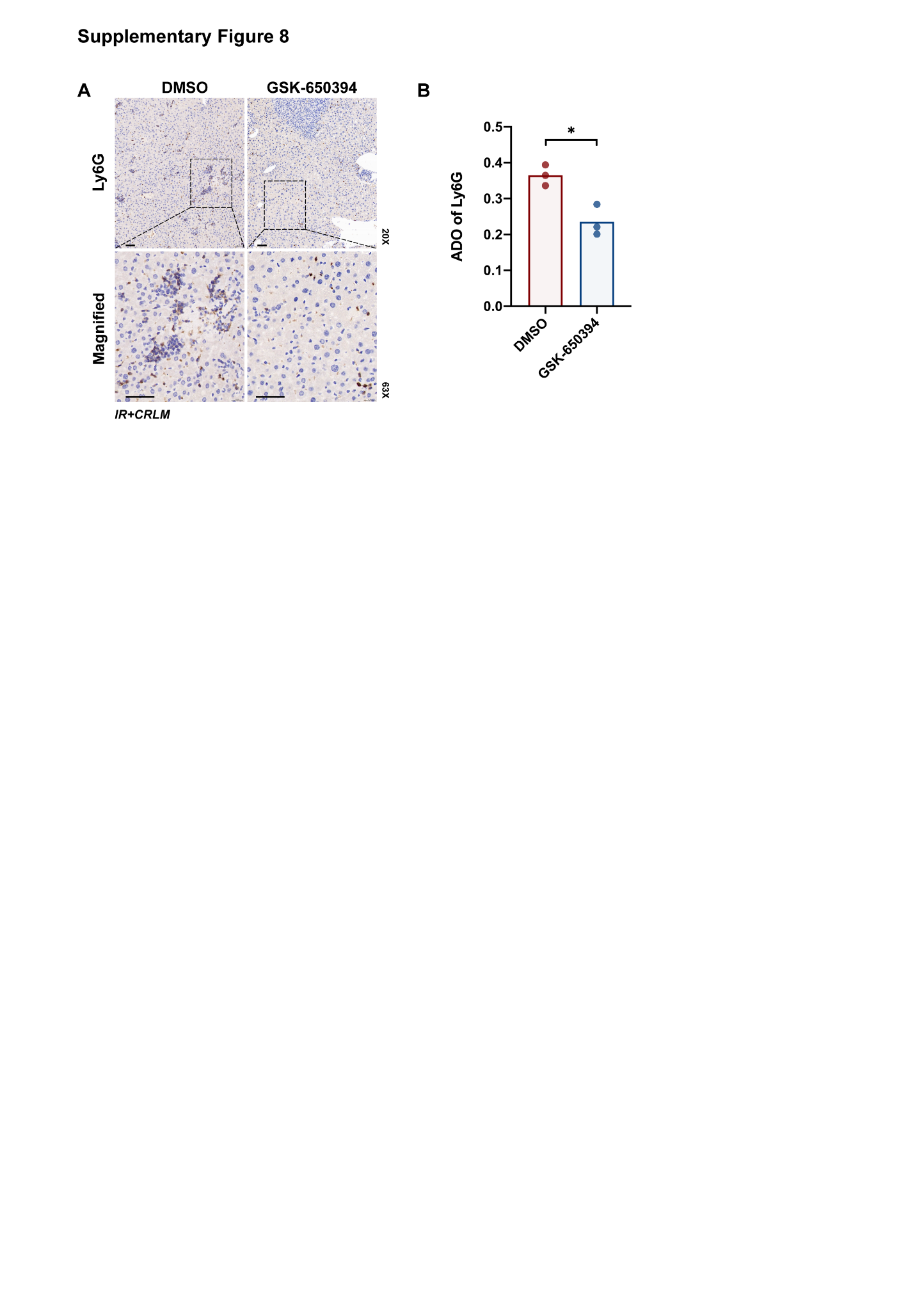


***Figure S8: Recruitment of Neutrophils in mice undergoing CRLM+IR is blocked by GSK-650394***

GSK-650394 was injected intraperitoneally into mice to construct a mouse CRLM+IR model, and the mouse livers were harvested two weeks later. (A) Immunohistochemical detection of ly6G, mice injected intraperitoneally with DMSO as control group, (n=3samples/group). Scale bars, 50μm. (B) Immunohistochemical quantification (n=3samples/group). All data represent the mean ± SD. *p < 0.05, **p < 0.01, ***p < 0.001, ****p < 0.0001.


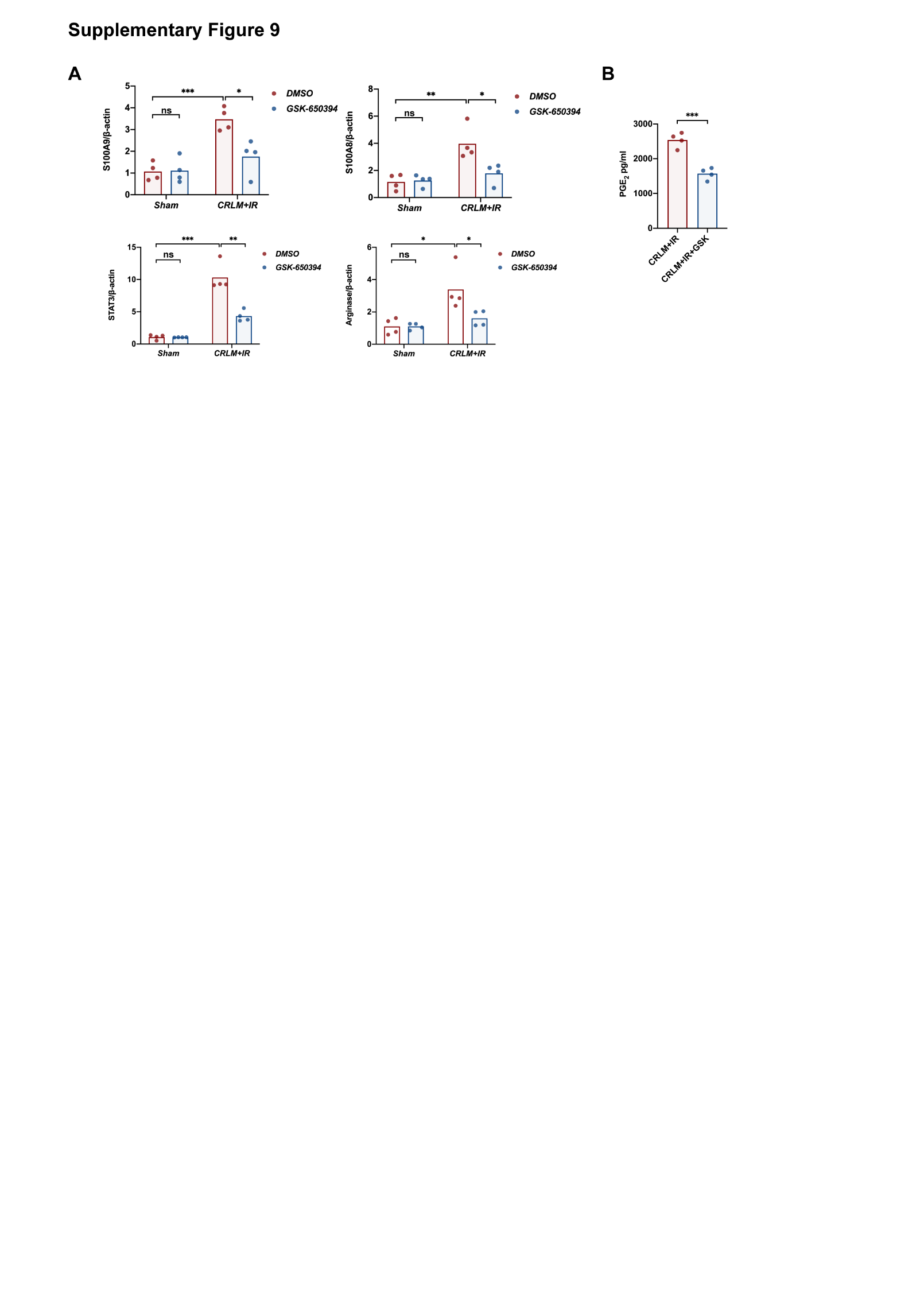


***Figure S9: GSK-650394 inhibits key biochemical signatures of PMN-MDSCs***

GSK-650394 was injected intraperitoneally into mice to construct a mouse CRLM+IR model. The livers and the serum were harvested two weeks later. (A) The mRNA level of S100A8、S100A9、ARG1、STAT3 in livers were detected by qPCR (n=4samples/group). (B) The expression of serum PGE2 and were detected by Elisa (n=4samples/group). All data represent the mean ± SD. *p < 0.05, **p < 0.01, ***p < 0.001, ****p < 0.0001.
